# Exploring new frontiers: cell surface vimentin as an emerging marker for circulating tumor cells and a promising therapeutic target in advanced gastric Cancer

**DOI:** 10.1186/s13046-024-03043-6

**Published:** 2024-04-30

**Authors:** Heming Li, Yang-Zhuangzhuang Zhu, Lu Xu, Tao Han, Jiasi Luan, Xin Li, Yuting Liu, Zhi Wang, Qiuge Liu, Xiangyu Kong, Chunpu Zou, Lin Su, Yifei Hou, Xiao Chen, Lujun Chen, Ruoyu Wang, Zihang Xu, Mingfang Zhao

**Affiliations:** 1 Department of Medical Oncology, The First Hospital of China Medical University, No.155 Nanjingbei Road, Shenyang, Liaoning 110001 People’s Republic of China; 2 Department of Oncology, Affiliated Zhongshan Hospital of Dalian University, Dalian, China; 3 Guangdong Association of Clinical Trials (GACT), Chinese Thoracic Oncology Group (CTONG) and Guangdong Provincial Key Lab of Translational Medicine in Lung Cancer, Guangzhou, Guangdong Province China; 4 School of Traditional Chinese Medicine, Shanghai University of Traditional Chinese Medicine, 1200 Cailun Rd., Pudong New District, Shanghai, 201203 China; 5 Key Laboratory of Structure-Based Drug Design & Discovery of Ministry of Education, Shenyang Pharmaceutical University, Shenyang, China; 6 The General Hospital of Northern Theater Command Training Base for Graduate, China Medical University, Shenyang, China

**Keywords:** Cell surface vimentin (CSV), Gastric cancer (GC), Circulating tumor cells (CTCs), Insulin-like growth factor-I receptor (IGF-IR), Fat mass and obesity-associated protein (FTO), Metastasis

## Abstract

**Background:**

Circulating tumor cells (CTCs) hold immense promise in guiding treatment strategies for advanced gastric cancer (GC). However, their clinical impact has been limited due to challenges in identifying epithelial-mesenchymal transition (EMT)-CTCs using conventional methods.

**Methods:**

To bridge this knowledge gap, we established a detection platform for CTCs based on the distinctive biomarker cell surface vimentin (CSV). A prospective study involving 127 GC patients was conducted, comparing CTCs enumeration using both EpCAM and CSV. This approach enabled the detection of both regular and EMT-CTCs, providing a comprehensive analysis. Spiking assays and WES were employed to verify the reliability of this marker and technique. To explore the potential inducer of CSV^+^CTCs formation, a combination of Tandem Mass Tag (TMT) quantitative proteomics, m^6^A RNA immunoprecipitation–qPCR (MeRIP–qPCR), single-base elongation- and ligation-based qPCR amplification method (SELECT) and RNA sequencing (RNA-seq) were utilized to screen and confirm the potential target gene. Both in vitro and in vivo experiments were performed to explore the molecular mechanism of CSV expression regulation and its role in GC metastasis.

**Results:**

Our findings revealed the potential of CSV in predicting therapeutic responses and long-term prognosis for advanced GC patients. Additionally, compared to the conventional EpCAM-based CTCs detection method, the CSV-specific positive selection CTCs assay was significantly better for evaluating the therapeutic response and prognosis in advanced GC patients and successfully predicted disease progression 14.25 months earlier than radiology evaluation. Apart from its excellent role as a detection marker, CSV emerges as a promising therapeutic target for attenuating GC metastasis. It was found that fat mass and obesity associated protein (FTO) could act as a potential catalyst for CSV^+^CTCs formation, and its impact on the insulin-like growth factor-I receptor (IGF-IR) mRNA decay through m^6^A modification. The activation of IGF-I/IGF-IR signaling enhanced the translocation of vimentin from the cytoplasm to the cell surface through phosphorylation of vimentin at serine 39 (S39). In a GC mouse model, the simultaneous inhibition of CSV and blockade of the IGF-IR pathway yielded promising outcomes.

**Conclusion:**

In summary, leveraging CSV as a universal CTCs marker represents a significant breakthrough in advancing personalized medicine for patients with advanced GC. This research not only paves the way for tailored therapeutic strategies but also underscores the pivotal role of CSV in enhancing GC management, opening new frontiers for precision medicine.

**Supplementary Information:**

The online version contains supplementary material available at 10.1186/s13046-024-03043-6.

## Background

Gastric cancer (GC) is a prevalent and aggressive malignant tumor that poses a substantial global health challenge [1]. Despite advancements in diagnosis and therapy, the overall survival (OS) and 5-year survival rates for patients with advanced GC remain poor and unsatisfactory [2, 3]. Hence, identifying precise biomarkers through non-invasive detection procedures is imperative for monitoring therapeutic responses and predicting disease relapse. Moreover, these biomarkers are crucial for the long-term management of advanced GC patients.

Liquid biopsy is a dynamic and rapidly evolving field within the realm of medical diagnostics, revolutionizing the way we detect, monitor, and manage various diseases, with a significant emphasis on cancer [4–6]. Circulating tumor cells (CTCs) represent a subset of these biomarkers that have attracted considerable attention in the field of liquid biopsy [7]. These cells, released from primary tumors or metastatic sites into the bloodstream, hold significant promise for diverse clinical applications. Nevertheless, our understanding of CTCs in advanced GC is limited due to the challenges posed by the epithelial-mesenchymal transition (EMT) process [8, 9]. EMT is a biological phenomenon through which cancer cells undergo transformative changes, rendering them more invasive, aggressive, and adept at evading immune system responses [10, 11]. The study of CTCs in advanced GC becomes more intricate due to the transformative effects of the EMT process on these cells. Such transformation has the potential to complicate the detection and analysis of CTCs [12]. Additionally, using markers such as EpCAM, vimentin, and N-cadherin for CTC detection is inaccurate, as these markers are also commonly expressed on leukocytes [13]. This challenge is even more pronounced when attempting to detect EMT-CTCs. To address this gap, it is crucial to identify new biomarkers specifically tailored to mesenchymal tumor cells in advanced GC patients.

Vimentin, a versatile intermediate filament protein recognized as a hallmark of EMT, plays a crucial role in various cellular processes [10]. Recent studies have shown its presence on the surface of cancer cells and its secretion by endothelial cells under specific conditions [13, 14]. This cell-surface vimentin (CSV) has gained attention due to its overexpression in cancer cells, opening doors for potential applications in the capture of CTCs across various tumor types, particularly in the context of advanced GC [15–18]. One promising avenue for targeting CSV is the use of monoclonal antibody 84-1. Our previous works have demonstrated its specific binding to CSV, offering a targeted approach for CTCs capture [15, 16]. However, it remains unclear whether CSV can serve as a universal biomarker for CTCs enrichment in GC patients. Furthermore, the mechanisms underlying the overexpression of CSV in cancer cells and its potential as a target for capturing CTCs in the context of CTCs generation are poorly understood.

In light of recent findings, it is evident that N6-methyladenosine (m^6^A) RNA methylation holds a pivotal role in the process of tumor metastasis by intricately regulating post-transcriptional gene expression [19]. Several studies have shed light on the significance of m^6^A methylation in this context. For instance, it has been demonstrated how m^6^A modifications can profoundly affect the stability and translation efficiency of mRNAs encoding genes involved in cancer progression [20, 21]. Additionally, a comprehensive analysis of m^6^A profiles has been conducted in various cancer types, revealing distinct patterns of m^6^A modification associated with metastatic potential, further underscoring the importance of this epigenetic mark in cancer metastasis [22, 23]. Notably, dysregulated m^6^A modification has been observed at significantly elevated levels in CTCs of lung cancer patients compared to whole blood cells [24]. Despite these valuable insights, our understanding of the precise role of m^6^A methylation in CTCs formation and metastasis in GC remains incomplete and necessitates further investigation. Therefore, it becomes imperative to explore the influence of m^6^A regulatory enzymes on the expression of critical genes in advanced GC patients to bridge this critical knowledge gap and expand our comprehension of the role of m^6^A in cancer metastasis.

In this study, we aimed to assess the effectiveness of CSV-positive-CTCs (CSV^+^CTCs) in comparison to the conventional marker EpCAM-positive-CTCs (EpCAM^+^CTCs) for detecting CTCs in both resectable and unresectable advanced GC patients. Our findings demonstrated the remarkable specificity of CSV in identifying CTCs, and further investigation revealed that FTO plays a pivotal role in the formation of CSV^+^CTCs, shedding light on its involvement in regulating GC cell metastasis. These discoveries underscore the potential of CSV as a valuable biomarker for early non-invasive cancer diagnosis, prognosis prediction, and as a tool to identify therapeutic targets for GC patients. In summary, detecting CSV^+^CTCs in advanced gastric cancer patients enables earlier, more accurate outcome predictions than traditional radiology, aiding in improved GC management and new therapy development. This approach offers improved long-term management for gastric cancer patients through less invasive methods, emphasizing its considerable benefits.

## Methods

The detailed procedures on methodology are presented under Supplemental Materials and Methods.

### Eligibility of patients and recruitment

This study was approved by the Institutional Review Board of Zhongshan Affiliated Hospital of Dalian University and The First Hospital of China Medical University (IRB Protocol: 2021011 and AF-SOP-07-1.1-01). Informed written consent was obtained from all enrolled patients. Peripheral blood samples were collected from 127 patients diagnosed with either resectable (pre-surgery) or unresectable (locally advanced and metastatic) GC at the Medical Oncology Department and Gastrointestinal Surgery Department. The resectable cohort consisted of GC patients scheduled for radical gastrectomy and D2 node dissection, while the unresectable cohort comprised patients with locally advanced tumors that were not amenable to surgical excision. The metastatic group comprised patients with confirmed distant metastasis. Patients with infections or secondary malignant diseases were excluded from the study. Additionally, blood samples were collected from healthy donors at the Healthy Examination Center. None of the healthy donors exhibited infections or signs of known malignant diseases at the time of blood collection.

### Study design

Clinicopathological information was recorded for each patient at the time of blood sample collection. The disease status of patients with unresectable or metastatic GC was assessed using the Response Evaluation Criteria in Solid Tumors (RECIST). This pilot study was aimed at the determination of CTCs. Blood samples were taken from patients with resectable GC before radical surgery, while for unresectable GC patients, blood samples were taken at the first time point of disease response evaluation on enrollment in this study. Based on the RECIST V1.1 criteria, the patients were categorized into responding (stable/partial response or complete response) and non-responding (progression) groups, depending on their evaluation outcome at the time of blood collection.

### Blood collection and processing

Peripheral blood (5 mL) was drawn from each enrolled participant, either before or at least 7 days after receiving intravenous anticancer drugs. Peripheral blood mononuclear cells (PBMCs) were prepared in line with established protocols. Blood was drawn into 10-mL BD Vacutainer tubes with K2 ethylenediamine tetra acetic acid (EDTA, BD Diagnostics), mixed at a 1:1 dilution with phosphate-buffered saline (PBS) containing 2% fetal bovine serum (FBS), and carefully layered over 3 mL of Ficoll-Paque PLUS density gradient medium (Ficoll-Paque PREMIUM) in a 15-mL SepMate tube (StemCell Technologies). The sample was centrifuged at 1200 g for 10 minutes at room temperature (RT) with the brake off. Thereafter, the PBMCs were collected from the top layer and washed twice with PBS containing 2% FBS at RT prior to the determination of CTCs.

### CSV^+^ or EpCAM^+^ cell detection

**Fig. 1:**
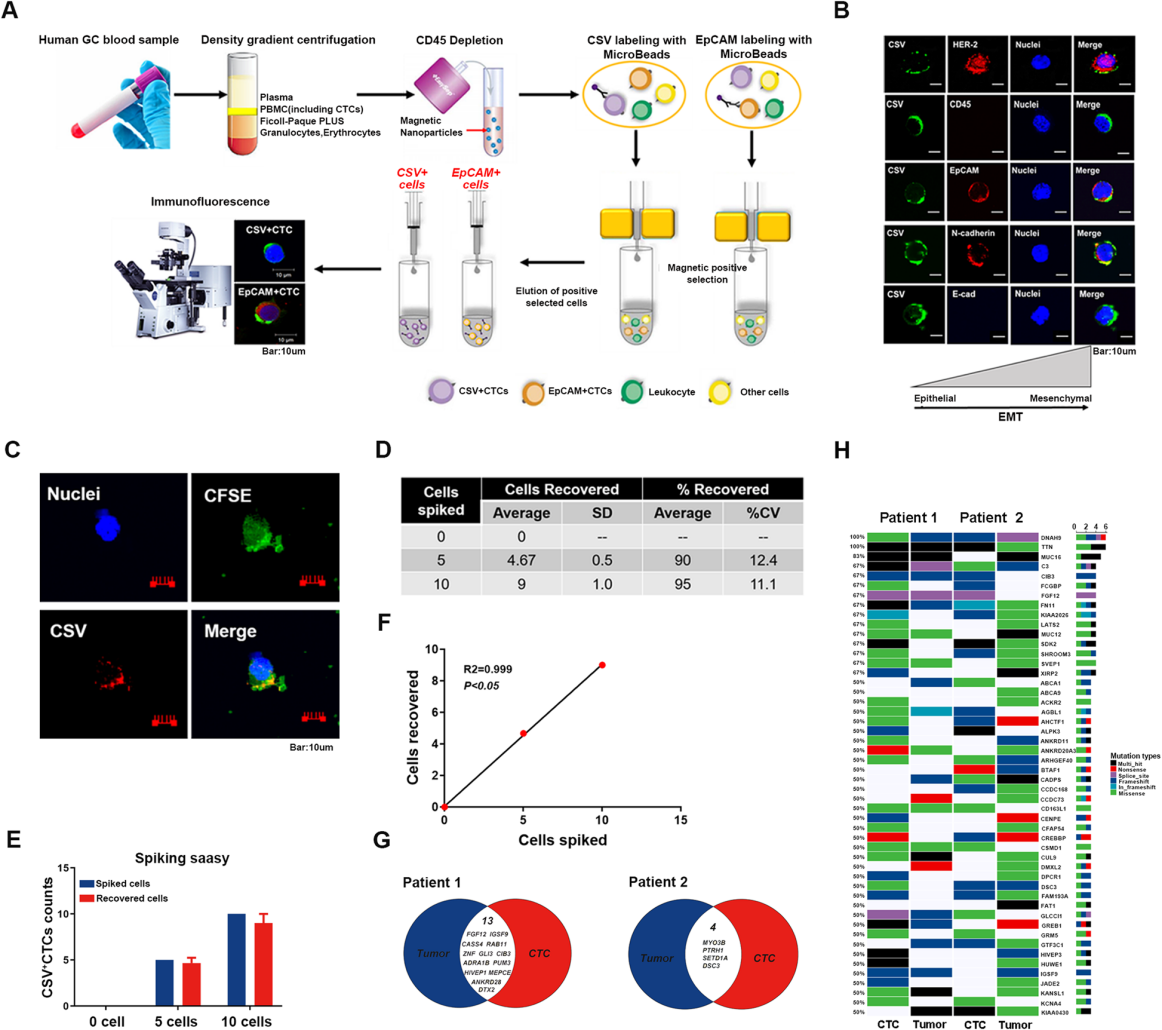
CTCs from GC peripheral blood samples are identified with high-sensitivity CSV antibody 84-1 and verified using spiking assays and WES. **A** The overall workflow of the CTCs detection technique using both the CSV and EpCAM markers. **B** Immunofluorescence staining for CSV (green), CD45, and EMT markers (red) in CTCs from a HER-2-positive GC peripheral blood sample. Scale = 10 μm. **C** Micrographs showing CFSE-labeled BGC-823 cells in peripheral blood mononuclear cells from healthy donors, CSV (84-1, red); tracker dye CFSE (green) and nuclear stain (blue). Scale = 10 μm. **D** & **E** Recovery rates for each concentration of spiked cells. The mean values were from at least three independent experiments (error bars indicate standard deviation). **F** Correlation of CTCs counts between the numbers of recovered cells and spiked cells. **G** WES analysis of CTCs isolated from two GC patients’ peripheral blood and matched tumor tissues. Venn diagrams showing common genes identified from paired tissue and CTCs. **H** Heatmap representation of the significantly mutated genes and mutation types from WES

The PBMCs were first depleted of CD45^+^ cells using the EasySep Human CD45 Depletion Kit (Stem Cell Technologies) in line with the manufacturer’s instructions. The resultant CD45^−^ cell populations were divided into two groups and subjected to selection for either CSV^+^ or EpCAM^+^ using the 84-1 or EpCAM antibody, respectively, along with mouse IgG-microbead (Miltenyi Biotec) binding. Then, the cells labeled with the appropriate antibody were isolated using a magnetic column according to the manufacturer’s protocol (Miltenyi Biotec). Subsequently, the CSV^+^CD45^−^ or EpCAM^+^CD45^−^ cells obtained were processed for further analysis (Fig. 1A).

## Results

### A CSV-based CTCs detection platform with high sensitivity and specificity

The previously established 84-1 CSV^+^CTCs identification method was used to enrich CTCs from peripheral blood samples of GC patients [15]. Figure 1A illustrates the overall workflow of the CTCs identification technique, involving the utilization of both CSV and EpCAM markers for the enrichment of cell populations (Fig. 1B). To evaluate the sensitivity of this innovative marker in identifying CTCs in GC patients, a blood spiking assay was performed using CFSE-labeled BGC-823 GC cells. The results demonstrated the ability of 84-1 antibody for identifying a low number of cells from PBMCs (Fig. 1C), with recovery rates exceeding 90% in 5- and 10-spiked cell groups (Fig. 1D and E). While the coefficient of variation increased as the number of spiked cells decreased (Fig. 1D), linear regression analysis revealed a positive correlation between the spiked cells and the recovered cells (*p* < 0.05; Fig. 1F). These findings indicate that the capturing of CSV^+^CTCs using this technique was highly sensitive for GC samples.

To verify the tumor origin of CTCs, CSV^+^CTCs were isolated from a peripheral blood sample of an advanced GC patient whose tumor tissue had been confirmed as HER2 positivity via IHC staining. Moreover, HER2 staining was conducted on the isolated CTCs (Fig. 1B, Supplementary Fig. S1). The results demonstrated that HER2 was highly expressed in the CTCs from this patient, indicating their tumor origin and the presence of an EMT phenotype. Additionally, whole-exome sequencing (WES) was performed on CTCs isolated from peripheral blood samples and matched tumor tissues of two advanced GC patients. Venn diagrams identified common genes from paired tissues and CTCs (Fig. 1G). Heatmap displayed significant gene mutations and their mutation types from WES (Fig. 1H). Analysis of intersecting genes in paired CTCs populations revealed that the CSV^+^CTCs were derived from GC tissues with high sensitivity and specificity. These results indicate that CSV holds significant potential as a biomarker for CTCs in GC patients.

### Comparison of data obtained from EpCAM and CSV isolation methods

**Fig. 2:**
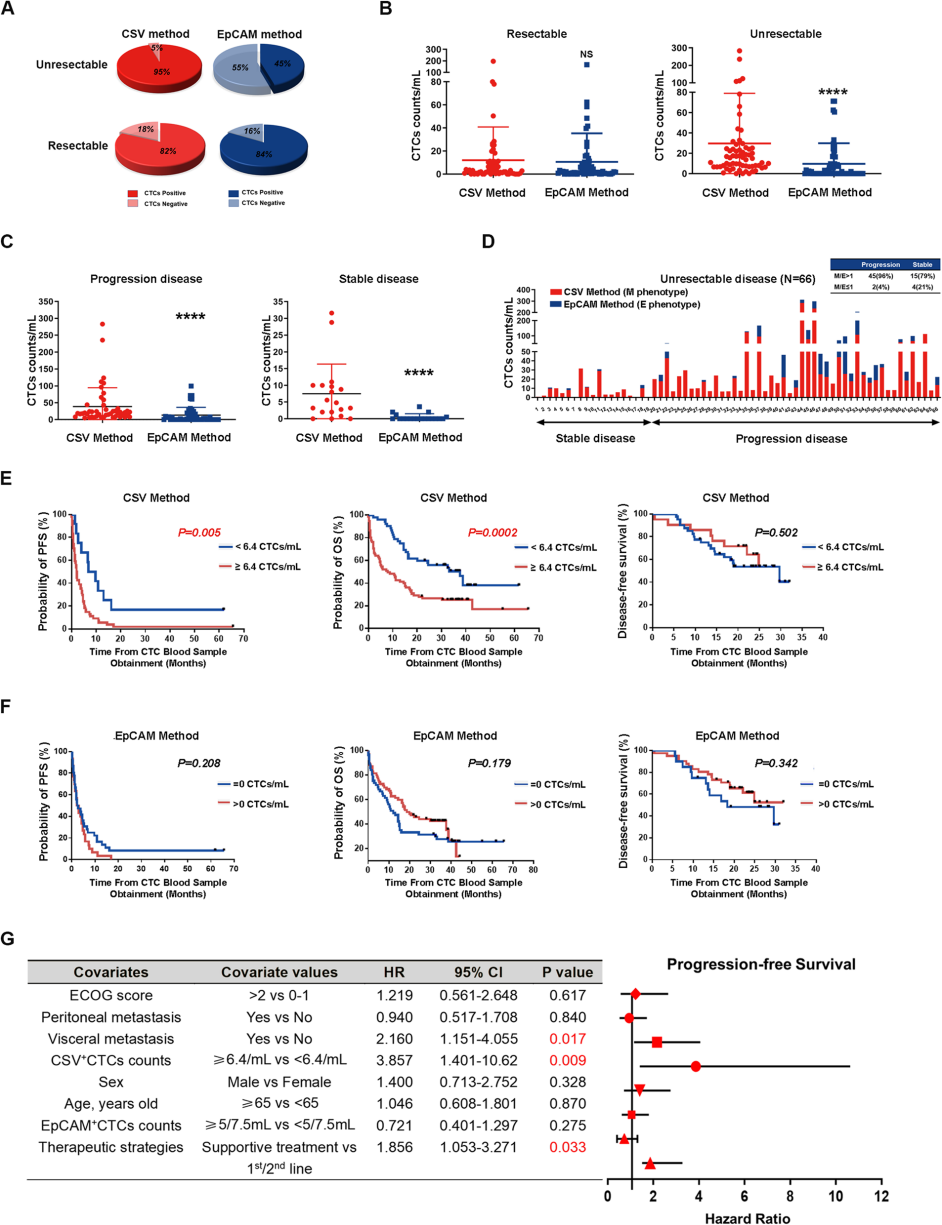
Comparison of data obtained from the EpCAM and CSV isolation methods. **A** Pie charts showing the positive detection of CTCs in GC patients with resectable and unresectable disease using both the CSV and EpCAM methods. **B** Comparison of CSV^+^CTCs counts and EpCAM^+^CTCs counts in patients with resectable GC or unresectable GC. **C** Progressive and stable categories of patients, based on clinical evaluations. Comparison of CSV^+^CTCs counts and EpCAM^+^CTCs counts in patients with stable or PD. NS = not significant; **** *P* < 0.0001. **D** Graphical representation of CTCs counts divided into epithelial (blue) and mesenchymal (red) phenotypes based on the method used for isolation from patients with stable disease or PD. **E** Kaplan-Meier curves of PFS (left), OS (middle), and DFS (right) for advanced GC patients according to CSV^+^CTCs counts, or (**F**) EpCAM^+^CTCs counts at baseline. **G** Subgroup analysis of PFS for advanced GC patients (ECOG = Eastern Cooperative Oncology Group; HR = hazard ratio)

Since EpCAM is a conventional marker for CTCs enrichment, we analyzed 127 blood samples from GC patients in this study using both CSV and EpCAM antibody methods to capture CTCs. Table 1 presents the clinical characteristics of GC patients. Additionally, 20 blood samples from healthy donors were tested for the presence of CSV^+^CTCs. The patients were categorized into resectable and unresectable disease groups, and the potential of CTCs in evaluating disease status was compared between these two isolation methods. As depicted in Fig. 2A, CSV^+^CTCs were detected in 95% of unresectable GC patients and 82% of resectable GC patients, whereas EpCAM^+^CTCs were observed in 45% of unresectable GC patients and 84% of resectable GC patients. The healthy donors did not show any detectable CTCs (data not shown), indicating a high specificity of the CSV 84-1 antibody. The correlation between CTCs counts and clinical features was presented in Table 2. Remarkably, the CSV method demonstrated a higher CTCs yield in samples from unresectable GC patients with advanced disease when compared to the EpCAM method, probably due to the association between increased CTCs counts and advanced-stage disease (Fig. 2A-B). The comparatively lower CTCs numbers detected by the EpCAM method may be attributed to the presence of EMT-CTCs, which remain undetectable using EpCAM-based assays.

**Table 1:** Clinical characteristics of GC patients (*N* = 127)

Clinical characteristic	***Number (%)***
Sex	
Male	90 (71%)
Female	37 (29%)
Age, years old	
<65	66 (52%)
≥65	61 (48%)
Disease status	
Resectable	61 (48%)
Unresectable	66 (52%)

**Table 2:** Correlation between CTCs counts and clinical features in both resectable and unresectable GC patients (*N* = 127)

Characteristic	CSV^**+**^CTCs			EpCAM^**+**^CTCs		
	Negative^a^	Positive^b^	*P* value	Negative^c^	Positive^d^	*P* value
Sex			0.092			0.118
Male	33 (26%)	57 (45%)		36 (28%)	54 (43%)	
Female	19 (15%)	18 (14%)		10 (8%)	27 (21%)	
Age, years old			0.104			0.278
<65	31 (24%)	35 (28%)		26 (20%)	40 (31%)	
≥65	21 (17%)	40 (31%)		20 (16%)	41 (33%)	
TNM			**<0.001**			**0.002**
I-II	20 (16%)	6 (5%)		3 (2%)	23 (18%)	
III-IV	32 (25%)	69 (54%)		43 (34%)	58 (46%)	
Metastasis			**<0.001**			**0.003**
Yes	8 (6%)	42 (33%)		26 (20%)	24 (19%)	
No	44 (35%)	33 (26%)		20 (16%)	57 (45%)	
Disease status			**<0.001**			**<0.001**
Resectable	40 (31%)	21 (17%)		10 (8%)	51 (40%)	
Unresectable	12 (9%)	54 (43%)		36 (28%)	30 (24%)	

The cutoff was defined based on ROC curves

^a^ CTCs counts <6.4/mL

^b^ CTCs counts ≥6.4/mL

^c^ CTCs counts =0/mL

^d^ CTCs counts > 0/mL

### CSV^+^CTCs is associated with therapeutic response in advanced GC patients

To investigate whether CTCs enumeration may serve as a predictor of therapeutic response in advanced GC patients using the both CSV and EpCAM methods, 66 patients with advanced-stage disease were categorized into responsive/stable disease (SD) and non-responsive/progressive disease (PD) groups, based on the Response Evaluation Criteria in Solid Tumors (RECIST) version 1.1 during blood sample collection. The findings of the correlation analysis between CTCs counts and clinical features in these patients were outlined in Table 3. A notably elevated positive percentage of CSV^+^CTCs was found to be significantly associated with high PS scores, older age and poor therapeutic response. The responsive/stable and non-responsive/progressive cohorts had higher CSV^+^CTC counts than EpCAM^+^CTC counts (Fig. 2C). As EpCAM is indicative of the epithelial subtype, while CSV is primarily associated with the mesenchymal type, we aimed to investigate whether the ratio of mesenchymal-to-epithelial content could serve as a potential indicator of therapeutic response. The enriched CTC subtypes were divided into epithelial (blue) and mesenchymal (red) fractions, based on EpCAM and CSV, respectively (Fig. 2D). Ratio-based analysis revealed that 45 out of 47 (96%) of advanced GC patients with a CSV/EpCAM ratio (M/E) of > 1 were in the PD group, while only 2 out of 47 (4%) of patients with M/E ≤ 1 were observed in this group (Fig. 2D). These findings further support the fact that a majority of CTCs enriched in the peripheral blood samples of advanced GC patients exhibit an EMT phenotype. Thus, CSV^+^CTCs was superior to EpCAM^+^CTCs in predicting therapeutic response in advanced GC patients and had improved sensitivity in determining the PD population.

**Table 3:** Correlation between CTCs counts and clinical features in unresectable GC patients (*N* = 66)

Characteristic	CSV^**+**^CTCs			EpCAM^**+**^CTCs		
	Negative^a^	Positive^b^	*P* value	Negative^c^	Positive^d^	*P* value
PS score			**<0.001**			**0.006**
0-1	11(16%)	9 (14%)		16(24%)	4 (7%)	
> 2	1 (2%)	45(68%)		20(30%)	26 (39%)	
Age, years old			**0.033**			0.099
< 65	9 (14%)	22(33%)		20(30%)	11 (17%)	
≥ 65	3 (5%)	32(48%)		16(24%)	19 (29%)	
Therapeutic strategies			0.625			0.597
Systemic therapy	6 (9%)	27(41%)		18(27%)	15 (23%)	
Supportive treatment	6 (9%)	27(41%)		18(27%)	15 (23%)	
Therapeutic response			**<0.001**			**0.042**
PR/SD	11(16%)	8 (12%)		14(21%)	5(8%)	
PD	1 (2%)	46(70%)		22(33%)	25 (38%)	
Visceral metastasis			0.188			0.311
Yes	6 (9%)	37(56%)		22(33%)	21(32%)	
No	6 (9%)	17(26%)		14(21%)	9 (14%)	

The cutoff was defined based on ROC curves

^a^ CTCs counts < 6.4/mL

^b^ CTCs counts ≥6.4/mL

c CTCs counts =0/mL

^d^ CTCs counts > 0/mL

### CSV^+^CTCs is associated with poor prognosis in advanced GC patients

To assess treatment effectiveness and prognosis, the cutoff values of 6.4 and 0 cells/mL for CSV^+^CTCs for EpCAM^+^CTCs, respectively, were determined through receiver operating characteristic (ROC) analysis. Unfavorable CSV^+^CTCs at baseline predicted inferior progression-free survival (PFS) and overall survival (OS) when compared to favorable CSV^+^CTCs at a median follow-up of 1.5 years (Fig. 2E). In contrast, EpCAM^+^CTCs did not show significant predictive values (Fig. 2F). Both CSV^+^CTCs and EpCAM^+^CTCs were not able to predict disease-free survival (DFS) in resectable GC patients during the early stage (Fig. 2E-F). Furthermore, Cox regression models indicated that the combination of CSV^+^CTCs with visceral metastasis and therapeutic strategies were independently associated with poor prognosis of advanced GC patients (Fig. 2G).

### CSV method is more sensitive and specific for evaluating GC patients with PD

**Fig. 3:**
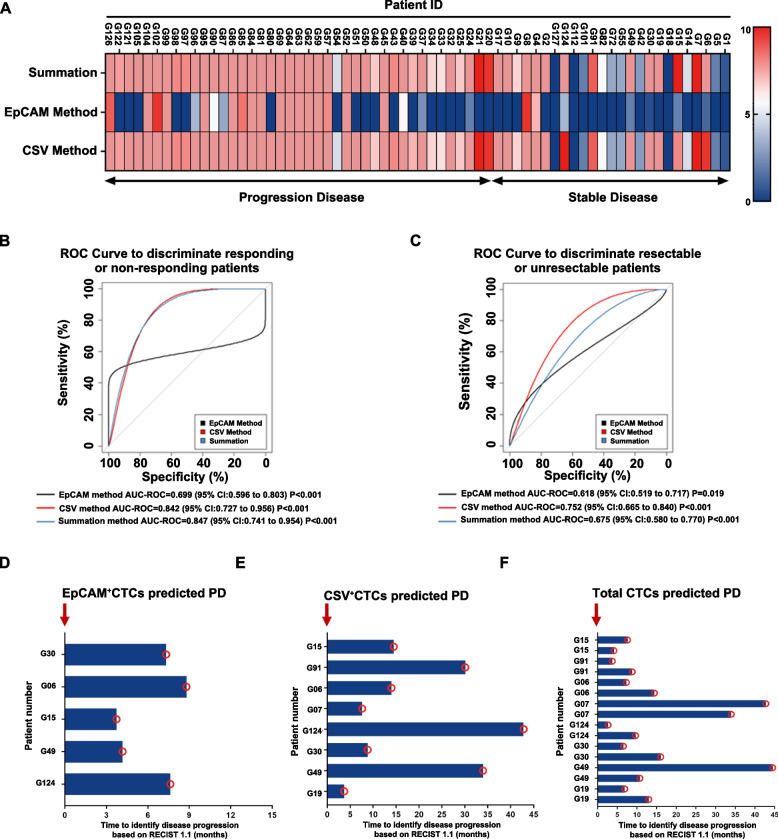
CTCs enumeration using both the EpCAM and CSV isolation methods. **A** Heatmap depicting the CTCs counts enumerated using the CSV method, EpCAM method, and a combination of the two methods with a gradient of CTCs counts from 0 (dark blue) to 10 (red) CTCs/mL. **B** ROC curves for CTCs counts using the CSV and EpCAM methods and a combination of the two methods, to distinguish patients with stable disease (responding) from those with PD (non-responding), and **(C)** advanced GC patients with resectable from unresectable disease. An AUC-ROC value closer to 1 denotes an ideal method for discrimination. **D** Column diagram showing the time gap between CTCs-predicted PD (arrow) and radiologically-predicted PD (circle) based on the EpCAM, **(E)** CSV, **(F)**, and a combination of the two methods

To assess the predictive efficacy of CTCs counts, we explored the possibility of combining the numbers obtained through both CSV and EpCAM marker identifications. Heatmap analysis (Fig. 3A) revealed that the combined method of CTCs counts could effectively predict disease outcomes in advanced GC patients. Additionally, we compared the correlations between therapeutic response and CTCs numbers determined with both markers via ROC curve analysis (Fig. 3B). The combined method of CTCs detection (AUC-ROC = 0.847) demonstrated higher sensitivity and specificity than the CSV-CTCs (AUC-ROC = 0.842) or EpCAM-CTCs (AUC-ROC = 0.699) detection method. However, the combined method (AUC-ROC = 0.675) did not exhibit superior discrimination of disease status compared to the CSV method (AUC-ROC = 0.752) (Fig. 3C). These findings indicate that the combined method is more sensitive and specific in predicting disease outcomes, but it may not exhibit superior performance evaluating GC patients with PD than those with stable disease compared to the CSV method.

### CTCs detection using the CSV method could predict therapeutic response earlier than radiology evaluation

RECIST criteria version 1.1 is the standard method for evaluating therapeutic response in solid tumors [25]. This study aimed to discover a novel way for predicting therapeutic efficacy earlier than RECIST 1.1 in GC patients receiving systemic therapy. After analyzing 16 advanced GC patients with positive CTCs detection, initially identified to have stable disease according to RECIST 1.1 during blood sampling, we compared the discrepancy between CTCs-predicted PD time and radiologically predicted PD time using different CTCs detection methods. The results showed that 31 and 50% of patients were not radiologically predicted to have PD at the time of EpCAM^+^CTCs and CSV^+^CTCs-predicted PD, respectively (Fig. 3D and E). More importantly, CTCs analysis using CSV-specific markers successfully predicted PD, with a median of 14.25 months earlier than radiology evaluation (Fig. 3E). In contrast, the median gap between EpCAM^+^CTCs-predicted PD and radiology-predicted PD was 7.33 months (Fig. 3D). This time gap extended to 9.24 months based on the PD predicted by the combined CTCs (Fig. 3F). Collectively, these results suggest that the use of CSV for CTCs enrichment may predict therapeutic response earlier than radiology evaluation.

### CSV expression is correlated with metastatic potential in GC

**Fig. 4:**
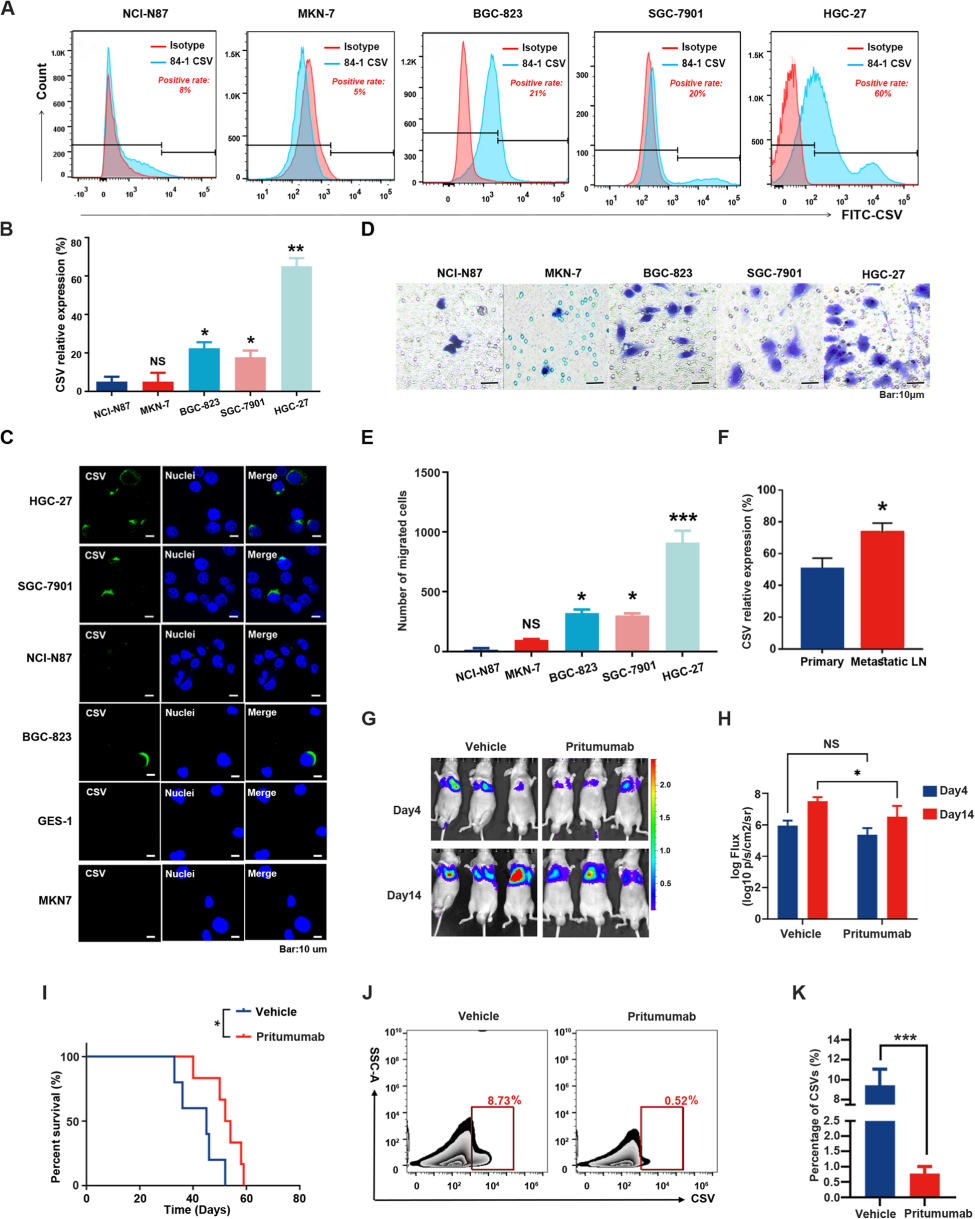
Correlation of CSV expression with metastatic potential in GC. **A** & **B** Immunological assessment of CSV expression in five GC cell lines by flow cytometry analysis. **C** Immunofluorescence staining of five GC cells for anti-CSV 84-1 (green) and nuclei (blue). Scale = 10 μm. **D** & **E** The migratory capacity of five GC cell lines, as compared with the N87 cell line measured using the Transwell chamber assay. The significant difference was compared with the N87 cell line. Scale = 10 μm. **F** The expression of CSV in primary tumor lesions and paired fresh metastatic lymph node samples from GC patients, as measured by flow cytometry analysis. **G** & **H** The lung metastasis mouse model established via injection of 5 × 10^6^ /100 μl 823-luc cells through the tail vein of the nude mouse, followed by daily treatment with 2 mg/kg Pritumumab. In vivo images taken on days 4 and 14 using an IVIS Lumina XR Imaging System. **I** The survival time of lung metastasis model mice in all groups. **J** & **K** CSV^+^CTCs derived from PBMCs of the lung metastatic model mice. * *P* < 0.05; ** *P* < 0.01; *** *P* < 0.001. NS = not significant

After establishing CSV as a marker for CTCs detection, we further investigated the correlation between CSV expression and metastatic potential in GC. A series of in vitro experiments were conducted on various GC cell lines using flow cytometry and immunofluorescence analyses. The findings revealed positive correlations among the migratory capacity of GC cells and CSV expression and EMT phenotype (Fig. 4A-E). Additionally, higher expression levels of CSV were observed in fresh metastatic lymph node samples, when compared to paired primary tumor lesions from GC patients (Fig. 4F), suggesting a potential association between CSV expression and the migratory capacity of GC cells.

To further evaluate the in vivo effect of CSV on GC metastasis, a lung metastasis mouse model was established by injecting 5 × 10^6^ /100 µl BGC-823 cells into three groups of nude mice. Unlike the antibody of 84-1, which is used for CTCs enrichment and immunofluorescence staining, Pritumumab is a therapeutic antibody that induces the death of glioma cells by binding to CSV, a novel approach in cancer treatment with unexplored efficacy in gastric cancer. Therefore, after confirmation of lung metastasis on day 4 using in vivo imaging, the model mice were intraperitoneally administered with Pritumumab, for 14 consecutive days. The results demonstrated the antitumor potential of Pritumumab, when compared to the control group (Fig. 4G and H). Moreover, all three groups of lung metastasis model mice exhibited significantly prolonged survival after treatment with Pritumumab (Fig. 4I). To further confirm the effect of Pritumumab on CSV in vivo, flow cytometry analysis was performed to determine CSV^+^CTCs derived from peripheral blood mononuclear cells (PBMCs) of the lung metastasis model mice. As shown in Fig. 4J and K, the population of CSV^+^CTCs was significantly reduced in the Pritumumab group compared to the control group. However, CSV^+^GC cells were not detected in subcutaneous xenografts, including both tumor tissues and PBMC (Supplementary Fig. S2). These results suggest that CSV expression is positively associated with GC metastatic abilities, and blocking of CSV expression with Pritumumab may serve as a novel pharmaceutical intervention for metastatic GC.

### The eraser gene of m^6^A RNA methylation (FTO) regulates CSV in advanced GC

Previous studies have demonstrated the significant role of m^6^A, a type of RNA modification, in the EMT process across various cancers. In this study, the impact of m^6^A on advanced GC and its relationship with vimentin expression were investigated. Specifically, m^6^A methylation patterns were analyzed in a well-annotated cohort of 300 GC patients from the Asian Cancer Research Group (ACRG). The analysis focused on two eraser genes (i.e., FTO and ALKBH5), and 716 differentially expressed genes (DEGs) associated with m^6^A were identified. The expression levels of these eraser genes in the m^6^A gene of the GSE62254 dataset were categorized into two clusters: EraserCluster A (ALKBH5 overexpression) and EraserCluster B (FTO overexpression) (Supplementary Fig. S3A and S3B). Three-dimensional visualization revealed clear segregation of the red and blue points into these two clusters (Supplementary Fig. S3C and S3D). Clinical relevance was further compared between EraserCluster A and EraserCluster B. Significant differences were observed in the distributions of ACRG subtype, stage, and histology between the two clusters (Supplementary Fig. S3C and Table S2). Based on the ACRG subtype classification, EraserCluster B was significantly associated with the EMT process. Notably, this cluster with FTO overexpression showed a high bias towards mid-to-late-stage (II/III/IV) status and poor prognosis (Supplementary Fig. S3A and S3C).

Gene ontology (GO) enrichment analysis identified 32 core targets primarily involved in cell signaling, adhesion, extracellular matrix organization, and cell proliferation, with both positive and negative regulation. Further gene set variation analysis (GSVA) revealed that EraserCluster B was associated with immune suppression and cancer-related biological processes. Subsequent analysis indicated a significant enhancement of stromal activity in EraserCluster B, including angiogenesis, EMT1, EMT2, EMT3, and Pan-fibroblast TGFβ (Supplementary Fig. S3E). These findings suggest that the eraser gene of m^6^A RNA methylation regulates the EMT process, with FTO being involved in CSV expression during EMT as well as CSV^+^CTCs formation.

### FTO promotes GC metastasis by regulating CSV expression

**Fig. 5:**
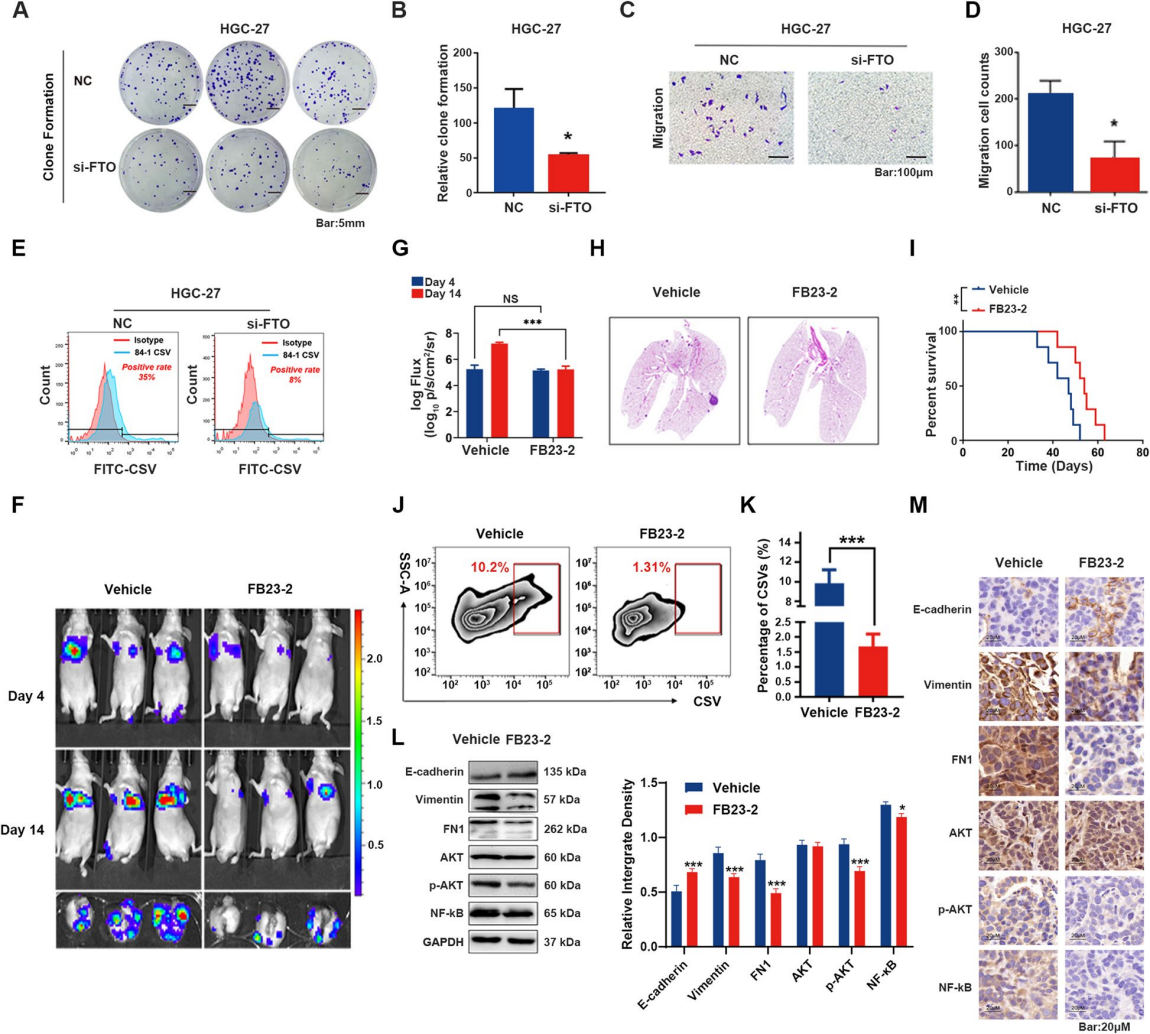
GC cell migration and metastasis are enhanced both in vitro and in vivo by FTO through regulation of CSV expression. **A** & **B** Knockdown of FTO impaired the colony-formation capacity of GC cells. **C** & **D** Knockdown of FTO decreased the invasion capacity in GC cells. **E** The expression levels of CSV in FTO knockdown GC cells, as assessed by flow cytometry analysis. **F** & **G** The antitumor effect of the FTO-specific inhibitor FB23-2 on lung metastasis model mice. A lung metastasis mouse model was established as described above, followed by daily treatment with DMSO (vehicle) or 4 mg/kg FB23-2 (intraperitoneally) for 2 weeks. In vivo images were taken on days 4 and 14 using an IVIS Lumina XR Imaging System. **H** GC metastases, as confirmed by H&E staining. **I** The survival time of lung metastasis model mice. **J** & **K** CSV^+^CTCs derived from PBMCs of the lung metastatic model mice, as determined by flow cytometry analysis. **L** Western blot analysis for determining the protein expression levels of E-cadherin, vimentin, FN1, p-AKT, AKT, and NF-kB in tumor tissues. **M** Expression levels of E-cadherin, vimentin, FN1, p-AKT, AKT, and NF-kB in tumor tissue sections, as determined by IHC staining. The results were examined under a microscope and captured (× 200). *P < 0.05; **P <0.01; *** *P* < 0.001; NS = not significant

To assess the effect of FTO on GC metastasis and development, experiments were conducted to silence FTO in HGC-27 cells. These results showed that either the knockdown of FTO or the treatment with the FTO-specific inhibitor, FB23-2 significantly reduced the metastatic and proliferative abilities of GC cells, as revealed by Transwell assay and the formation of cell clones (Fig. 5A-D and S4). Moreover, the expression levels of CSV were significantly decreased in HGC-27 cells with FTO knockdown compared to the negative control group (8% vs. 35%; Fig. 5E). To further verify the in vivo effect of FTO on GC metastasis, a lung metastasis mouse model was established. Lung metastasis was confirmed using in vivo imaging on day 4, after which the FTO-specific inhibitor, FB23-2, was intraperitoneally injected into the model mice. The results demonstrated that the FB23-2 group exhibited anti-metastatic activity, leading to prolonged survival (Fig. 5F-I). This may be attributed to the inhibition of CSV^+^CTCs formation and reversed EMT process in the GC mouse model (Fig. 5J-M). Further, our in vivo studies highlighted the absence of CSV^+^GC cells in subcutaneous xenografts, unlike their clear presence in lung metastasis models (Supplementary Fig. S2). This distinction underscores the role of CSV in promoting cancer metastasis through the bloodstream, given its known association with metastatic potential.

### Identification of potential target genes of FTO in GC

**Fig. 6:**
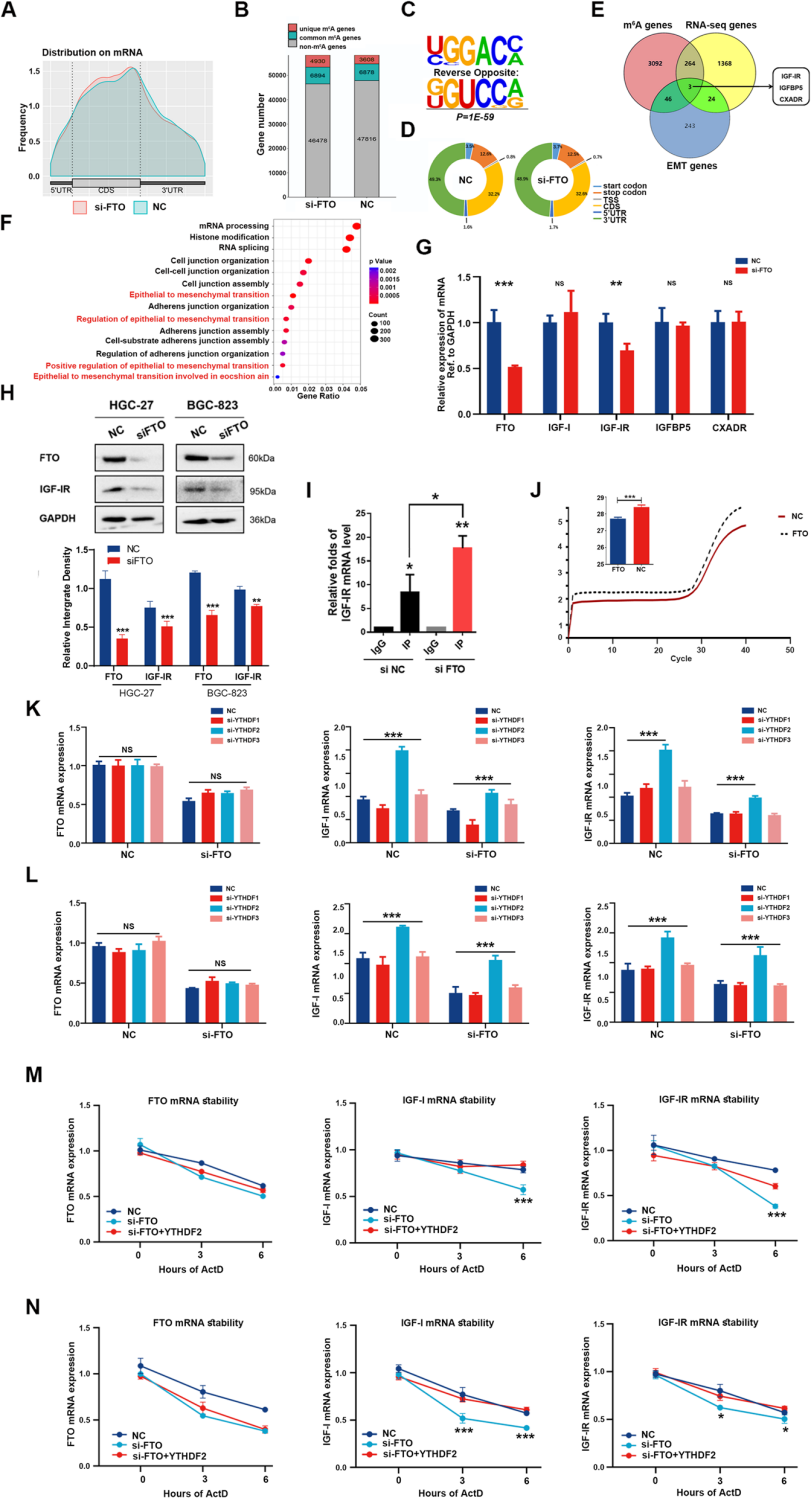
IGF-IR is a target gene of FTO-mediated m^6^A modification. **A** Distribution of m^6^A peaks across mRNA transcripts. The 5′-untranslated region (5′-UTR), coding region (CDS), and 3′-untranslated region (3′-UTR) divided into 100 segments, and the percentages of m^6^A peaks that fall within each segment are shown. **B** The number of m^6^A-modified genes identified by m^6^A-seq was compared between control and si-FTO HGC-27 cells. Common m^6^A genes were defined as having at least one common m^6^A peak, while unique m^6^A genes were defined as having no shared common m^6^A peaks. **C** Top consensus m^6^A motif identified with m^6^A peaks in HGC-27 cells with FTO knockdown. **D** The proportion of m^6^A peak distribution in the indicated regions across the entire set of mRNA transcripts between control and si-FTO HGC-27 cells. **E** Venn diagram showing overlapped transcripts with increased m^6^A peaks and differentially expressed genes based on RNA-seq and m^6^A-seq after FTO knockdown and EMT-related functional genes. **F** The significantly enriched GO terms in the biological process category for down-regulated genes from HGC-27 cells with FTO knockdown. **G** qPCR analysis for determining the mRNA expression levels of genes in HGC-27 cells with NC or si-FTO. **H** Western blot analysis of IGF-IR in HGC-27 and BGC-823 cells with or without FTO knockdown. **I** MeRIP-qPCR analysis of IGF-IR m^6^A level in NC or si-FTO HGC-27 cells. **J** CT values of IGF-IR with m^6^A modifications, as determined using the FTO-assisted SELECT m^6^A detection method. **K** & **L** qPCR analysis for determining the mRNA expression levels of FTO (left), IGF-I (middle), and IGF-IR (right) in HGC-27 cells (**K**) and BGC-823 cells (**L**) with or without FTO knockdown, in combination with siRNA knockdown of the m^6^A readers YTHDF1-3. **M** & **N** qPCR analysis of the mRNA stability of FTO (left), IGF-I (middle), and IGF-IR (right) in HGC-27 cells (**M**) and BGC-823 cells (**N**) with or without FTO knockdown in combination with or without si-YTHDF2. * *P* < 0.05; ** *P* < 0.01; *** *P* < 0.001; NS = not significant

To identify the potential target genes of FTO in GC, RNA-Seq analysis was performed to compare gene expression profiles after FTO knockdown in HGC-27 cells (Supplementary Fig. S5). Subsequently, m^6^A sequencing (m^6^A-seq) was conducted to map the variations of m^6^A modification in FTO knockdown GC cells and determine whether the altered gene expression was associated with FTO-regulated m^6^A modification. The m^6^A-seq analysis revealed that knockdown of FTO increased m^6^A enrichment in coding sequence (CDS) regions and 3′-untranslated regions (UTRs), but the effects on 5′-UTRs were minimal (Fig. 6A). Moreover, a total of 3608 and 4930 unique m^6^A genes were identified in control and FTO knockdown cells, respectively (Fig. 6B). The GGAC motif was found to be highly enriched within m^6^A sites in FTO knockdown cells (Fig. 6C), and both total and unique peak distribution patterns were altered (Fig. 6D). To identify targets involved in m^6^A-regulated EMT, further analyses were performed on 316 EMT-related genes, 3405 m^6^A-regulated genes, and 1659 altered genes, based on RNA-Seq data (with > 1.5-fold m^6^A change). Notably, three overlapping genes were identified, and IGF-IR was selected as a key factor for EMT, based on previous literature [26–29] (Fig. 6E). Gene ontology analysis of these genes revealed their potential associations with cell junctions and EMT regulation (Fig. 6F).

Furthermore, the downregulation of IGF-IR was confirmed through sequencing data (Fig. 6G-H). To investigate the role of FTO in m^6^A modification of IGF-IR mRNA, the m^6^A-sequencing data was validated using both MeRIP-qPCR and the Epi-SELECTTM m^6^A fraction quantification kit (Epibiotek) method. The results demonstrated that anti-m^6^A antibody significantly enriched the mRNA levels of IGF-IR in FTO knockdown GC cells (Fig. 6I), and there were significant decreases in the CT values of IGF-IR with m^6^A modifications after FTO demethylation treatment (Fig. 6J). Additionally, analysis of public databases revealed high expression levels of FTO in pan-tumors, with a positive correlation between the FTO and IGF-IR levels and poor prognosis (Supplementary Fig. S6). Collectively, these data suggest that IGF-IR is a potential target gene of FTO in GC.

### YTHDF2-mediated RNA decay regulates the expression of FTO’s target genes

An investigation was conducted to examine how YTHDF family members influence the regulatory role of FTO in modulating IGF-IR expression through m^6^A RNA modification. As shown in Fig. 6K and L, knockdown of YTHDF1 or YTHDF3 had no effects on IGF-IR or IGF-I mRNA levels in both control and FTO-knockdown GC cells. However, the knockdown of YTHDF2 significantly increased the mRNA expression of both IGF-I and IGF-IR in the control group, but this effect was reversed in FTO-knockdown GC cell lines (Fig. 6K-L and Supplementary Fig. S7). Furthermore, FTO knockdown reduced the stability of IGF-I and IGF-IR mRNAs, while knockdown of YTHDF2 increased IGF-IR mRNA stability in FTO-knockdown GC cells (Fig. 6M-N). These findings suggest that YTHDF2-mediated mRNA decay plays a crucial role in regulating the expression of the potential target genes of FTO (i.e., IGF-IR and IGF-I) in GC cells.

### Further identification of IGF-IR as a direct target of FTO through molecular docking and molecular dynamics simulation

**Fig. 7:**
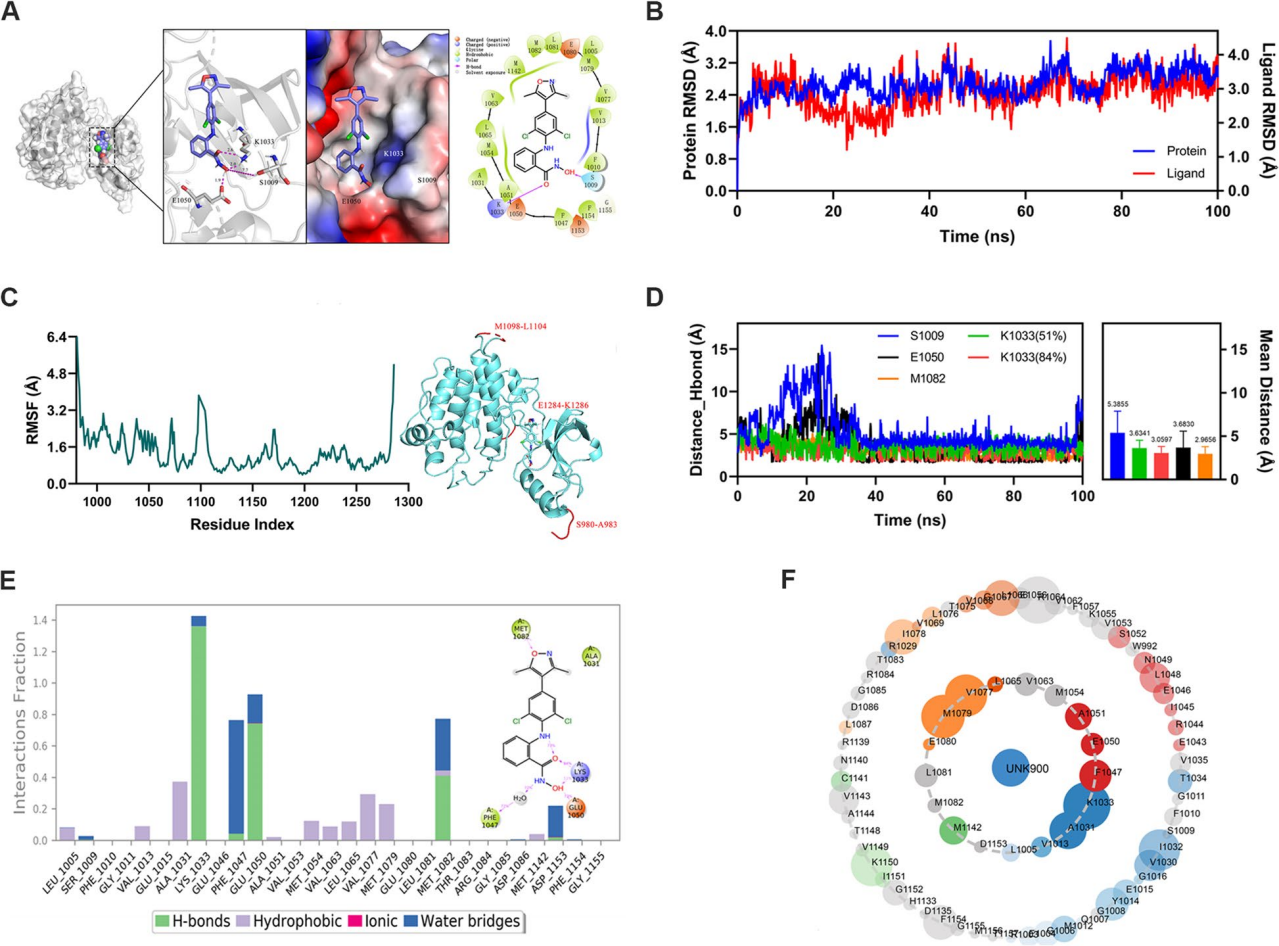
Further identification of IGF-IR as a direct target of FTO by molecular docking and molecular dynamics simulation. **A** 3D binding mode of complex (left), overall binding of the FTO-specific inhibitor FB23-2 within IGF-IR pocket (middle), and 2D binding mode of complex (right). **B** RMSD plot of IGF-IR backbone over a course of 100 ns simulation. **C** RMSF plots of IGF-IR and FB23-2 complex over MD simulation with flexible loops labeled in red. **D** Distances between FB23-2 and crucial residues during MD simulation. **E** Interaction fraction of IGF-IR key residues during MD simulation. **F** Asteroid plots of the complex

To elucidate the mechanism of binding between the FTO-specific inhibitor FB23-2 and IGF-IR, molecular docking was used to analyze the ligand-protein interaction. The results showed an RMSD value of 1.168 Å when the IGF-IR co-crystalline compound was re-docked within the active site of IGF-IR, indicating the reliability of the molecular docking methodology. Figure 7A illustrates the docking mode for FB23-2 against IGF-IR, where the residues SER1009 and LYS1033 of IGF-IR formed hydrogen bonds with FB23-2. The N-hydroxy benzamide group of FB23-2 fitted perfectly into the pocket generated by GLU1050 and LYS1033, thereby leading to a strong interaction with IGF-IR.

To further confirm the stability and behavior of the IGF-IR and FB23-2 complex, 100 ns of molecular dynamics (MD) simulations were carried out. It was found that the RMSD curves of the protein backbone and ligand were stable, with fluctuations around 2.6 Å and 2.9 Å, respectively (Fig. 7B). This indicates that the binding mode remains reliable throughout the simulation. Additionally, the RMSF plot of the IGF-IR with FB23-2 complex indicated that the fluctuations were in the flexible loop region (indicated in red letters), suggesting a strong affinity of FB23-2 towards IGF-IR (Fig. 7C). Collectively, the stability of the complex, consistent binding mode, and the observed fluctuations in the flexible loop region support the notion that FB23-2 forms a stable and reliable interaction with IGF-IR, indicating a strong binding affinity.

The interaction fraction was also analyzed for the IGF-IR/FB23-2 complex. Figure 7E demonstrates that FB23-2 could interact with several amino acids, such as LYS1033, GLU1050, MET1082, and PHE1047, through hydrogen bonds and water bridge interactions, thereby contributing to 84, 73 and 40% of the total simulation process, respectively. Moreover, the Asteroid diagrams (Fig. 7F) indicated that the residues LYS1033, GLU1050, and MET1082 of IGF-IR were crucial for maintaining a favorable H-bond distance with FB23-2 during the MD simulation, thereby significantly contributing to its interaction with the compound. Conversely, SER1009 exhibited an unstable molecular interaction (Fig. 7D-F). These findings indicate the robust binding affinity of FTO towards IGF-IR, which provides further support for the identification of IGF-IR as a direct target gene of FTO.

### Significance of IGF-IR signaling pathway in regulating CSV expression

Previous research demonstrated the involvement of the IGF-I/IGF-IR signaling pathway in GC during the EMT process. Gene set enrichment analysis (GSEA) revealed a positive enrichment of EMT progression in gene sets related to the IGF-I/IGF-IR signaling pathway (NES = 1.82; FDR = 0.052; Supplementary Fig. S8A and S8B). These findings further suggest that the IGF-I/IGF-IR signaling pathway may contribute to the regulation of CSV expression and CSV^+^CTCs formation in GC patients.

To investigate the regulatory role of the IGF-IR signaling pathway on vimentin expression, TMT quantitative proteomics analysis was conducted on HGC-27 GC cell lines stimulated with IGF-I for EMT induction (Supplementary Fig. S9-S10). The results revealed significant increases in the expression levels of 33 EMT-associated proteins in the IGF-I stimulation group (Supplementary Fig. S10A). Furthermore, GO enrichment analysis revealed that these differentially expressed proteins were primarily involved in EMT, membrane cadherin binding, and growth factor binding (Supplementary Fig. S10B). Subsequent analysis identified 15 key IGF-I-related pathways, including the MAPK and NF-kappa B signaling pathways, which exhibited significant enrichment (Supplementary Fig. S10C). To enhance the visualization of the results, Hallmark “wheels” with color-coding was used, based on negative log-transformed *p*-values obtained from the hypergeometric test. Among the differentially expressed proteins in IGF-I stimulation groups, 7 proteins were found to interact directly with IGF-IR signaling through protein-protein interactions (PPIs). These proteins included vimentin (VIM), CTNNB1, LASP1, PPP1CA, PAK2, RAB7A, and TWIST1 (Supplementary Fig. S10D). These results provide further support for the critical role of the IGF-IR signaling pathway during the EMT process as well as the regulation of vimentin expression.

### IGF-I-induced vimentin phosphorylation and translocation enhance CSV expression in GC cells

**Fig. 8:**
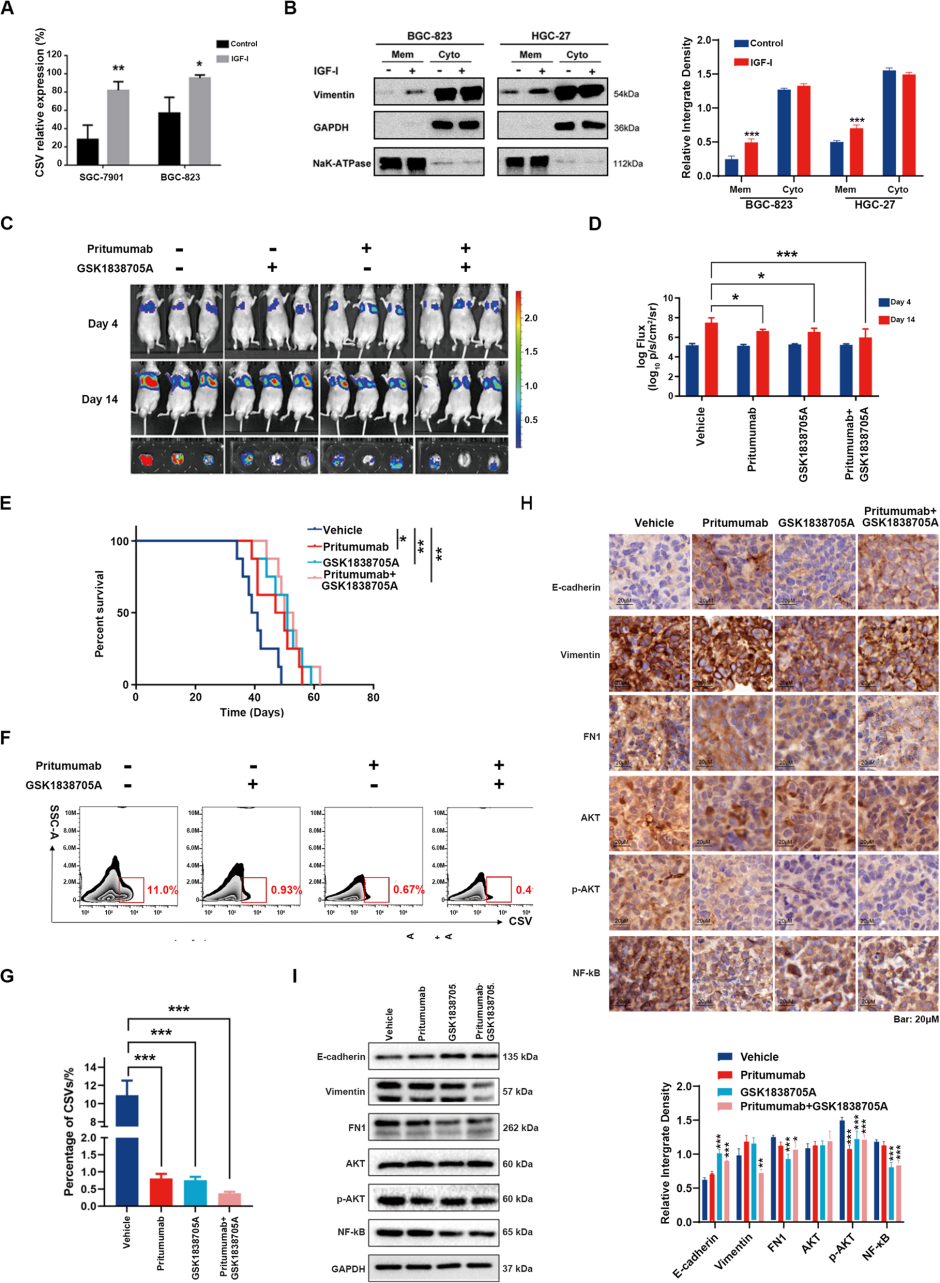
Blocking CSV in combination with an IGF-IR inhibitor represses CSV^+^CTCs and lung metastasis in nude mice. **A** GC cells serum-starved overnight and treated with or without 100 ng/mL IGF-I for 48 h. The expression levels of CSV, as assessed by flow cytometry analysis. **B** Western blot analysis of vimentin in cell lysates harvested from the cell membrane and cytoplasm. Na^+^-K^+^-ATPase was selected as an internal control for membrane proteins. **C** & **D** A lung metastasis mouse model was established via injection of 5 × 10^6^ /100 μl 823-luc cells through the tail veins of nude mice, followed by daily treatment with 2 mg/kg Pritumumab, 4 mg/kg GSK1838705A, or their combination for 2 weeks. In vivo images taken on days 4 and 14 using an IVIS Lumina XR Imaging System. **E** The survival time of lung metastasis model mice in all groups. **F** & **G** Flow cytometry showing the number of CSV^+^CTCs in the blood samples of model mice. **H** Expression levels of E-cadherin, vimentin, FN1, p-AKT, AKT, and NF-kB in tumor tissue sections, as determined by IHC staining. The results were examined under a microscope and photographed (× 200). **I** Western blot assay showing the protein expression levels of E-cadherin, vimentin, FN1, p-AKT, AKT, and NF-kB in tumor tissues. NS = not significant; * *P* < 0.05; ** *P* < 0.01; *** *P* < 0.001

Flow cytometric analysis revealed that activation of the IGF-IR signaling pathway is a critical regulator of CSV expression. The results demonstrated significant increases in the levels of vimentin bound to the surfaces of GC cells following stimulation with IGF-I (*p* < 0.05, Fig. 8A). To elucidate molecular mechanisms underlying the upregulation of CSV in GC cell lines, the subcellular localization of CSV was determined. Interestingly, after 48 hours of IGF-I stimulation, there was a noticeable increase in the expression levels of vimentin on the cell membrane (Fig. 8B). Because phosphorylation significantly impacts protein function and location, we further explored how vimentin moved from the cytoplasm to the membrane by identifying several serine phosphorylation sites. Our findings showed that activating IGF-I/IGF-IR signaling leads to vimentin phosphorylation at serine 39 (S39). This phosphorylation could be reversed by the IGF-IR-specific inhibitor GSK1838705A, as shown in Supplementary Fig. S11A-B. Additionally, HGC-27 GC cells transfected with a plasmid expressing either the wild type (WT) or the phosphor-dead mutant S39A were examined for CSV expression levels via flow cytometry. Relative to the wild-type control, the continuous S39 dephosphorylation mimicked by the S39A mutation had little impact on the CSV upregulation under IGF-I treatment (Supplementary Fig. S11C). These above findings indicated that CSV, a specific marker of EMT, was induced by IGF-I, and undergone translocation from the cytoplasm to the cell surface through vimentin phosphorylation in GC cells. Hence, the activation of the IGF-IR signaling pathway might lead to the upregulation of CSV in GC cells by facilitating the translocation of vimentin from the cytoplasm to the cell surface.

### Repression of CSV in combination with IGF-IR inhibitor suppresses CSV^+^CTC formation and metastasis in nude mice

To further validate the findings from in vitro experiments, an in vivo study was performed using a lung metastasis mouse model as previously described. The combination of Pritumumab and GSK1838705A was chosen due to their distinct mechanisms of action, aiming for a synergistic therapeutic approach. On day 4, after confirming lung metastasis through in vivo imaging, the model mice were treated with the blocking CSV antibody Pritumumab via intraperitoneal injection, the IGF-IR inhibitor GSK1838705A via intragastric administration, or a combination of both treatments, for 14 consecutive days. Comparisons were made with a control group. Both Pritumumab and GSK1838705A exhibited similar anti-tumor effects compared to the control group, but the combination of Pritumumab and GSK1838705A exhibited a significantly better effect (Fig. 8C and D; Supplementary Fig. S12). Furthermore, all three treatments significantly prolonged the survival rates of mice with lung metastasis, when compared to the control group. Specifically, the group that received both Pritumumab and GSK1838705A had the longest survival (Fig. 8E).

To further confirm the in vivo effects of Pritumumab and GSK1838705A on CSV expression, flow cytometry analysis was performed to determine the CSV^+^CTC populations derived from PBMCs from the lung metastasis model mice. As shown in Fig. 8F and G, the number of CSV^+^CTCs was significantly reduced in the Pritumumab, GSK1838705A, and Pritumumab+GSK1838705A treatment groups compared to the control group. Moreover, an IHC staining assay of lung tumor tissues revealed increased expression of E-cadherin and a corresponding decrease in the expression levels of vimentin and fibronectin (FN1) (Fig. 8H), indicating a potential inhibitory effect on EMT. The activation of downstream signaling pathways of IGF-I, as predicted from the results of TMT quantitative proteomics (Supplementary Fig. S10), was evaluated using IHC and Western blot assay. The expression levels of p-AKT and NF-κB were significantly inhibited in the Pritumumab, GSK1838705A, and combination treatment groups compared to the control group. These results were further confirmed by Western blot analysis (Fig. 8I). Altogether, these data support the crucial role of IGF-IR-mediated CSV upregulation in enhancing GC metastasis.

## Discussion

Recent advances in liquid biopsies and NGS sequencing technology have revolutionized cancer treatment, leading to FDA-approved strategies for the detection and management of cancer patients [4, 6]. However, despite efforts to improve liquid biopsies by isolating CTCs, their clinical utility in GC patients is limited. Therefore, it is of great importance to identify new biomarkers for non-invasive prediction of therapeutic efficacy and long-term prognosis. Furthermore, the molecular mechanisms underlying CTC formation are not well-understood, impeding the development of targeted therapeutic approaches. To address these gaps, a prospective study was conducted to compare the populations of CTCs in GC patients using EpCAM, and the corresponding population using CSV-specific antibody 84-1, a validated universal marker for identifying EMT-CTCs in various tumor types. The findings revealed that CSV^+^CTCs were the predominant CTC population, thereby providing a more accurate assessment of therapeutic efficacy and prognosis in advanced GC patients. Notably, this study identified the m^6^A demethylase FTO as a crucial regulator of CSV^+^CTC formation and metastasis by inhibiting IGF-IR mRNA decay and facilitating vimentin translocation from the cytoplasm to the cell surface. Hence, implementing the CSV^+^CTC detection method provides new perspectives for evaluating relapse risks and tailoring personalized medicine approaches in patients with advanced GC.

CTCs, also known as “liquid biopsies”, play a vital role in enhancing the effectiveness of cancer diagnosis, prognosis, and treatment [7]. Monitoring CTCs before and after treatment facilitates early diagnosis of subclinical patients or disease relapse, as well as prediction of tumor progression [8]. Various methods have been explored for CTCs isolation in GC, including immunomagnetic separation, microfluidic devices, and size-based filtration. The common method for identifying CTCs in gastrointestinal and colorectal cancers involves immunofluorescence assays. These assays use specific biomarkers such as cytokeratins (CKs) for epithelial cells, vimentin for mesenchymal cells, and CD45 for hematopoietic cells to define different CTC types [30]. Some studies have focused on the expression of specific markers such as EpCAM to capture CTCs, while others have investigated the role of CTC clusters. Notably, the CellSearch System, relying on antibody-based methods, has demonstrated remarkable success, and it is currently the only FDA-approved CTC detection method. However, identifying CTC populations in GC patients remains challenging due to their EMT features and low expression of the EpCAM marker [30]. This limitation hampers understanding of disease progression in advanced and metastatic GC cancer, which is crucial for selecting appropriate treatment strategies. Although numerous studies have investigated different mesenchymal biomarkers used to assess EMT-CTC phenotypes, no specific or universal biomarker has been identified.

EMT plays a critical role in GC progression and metastasis [11]. EMT-derived cells are associated with the induction of CTCs in the bloodstream, subsequently colonizing distant organs and forming metastases [7]. However, to date, no specific markers for EMT-CTCs have been identified in advanced GC patients. Vimentin, an intermediate filament protein integral to vimentin adhesion networks, has emerged as a significant EMT marker associated with tumor metastasis [31]. Recent research has demonstrated a subset of aggressive CTCs that express vimentin on their surface and exhibit an EMT phenotype, increasing the likelihood of tumor recurrence [18, 32]. To isolate these EMT-CTC cells, researchers have identified a promising marker called CSV. The CSV-specific monoclonal antibody, 84-1, has been developed and validated as a promising tool for enriching CTCs from metastatic and relapsed patients, outperforming the CellSearch method [18]. For instance, several researchers introduced the use of CSV as a marker for detecting mesenchymal CTCs in prostate cancer patients [33]. Their findings suggest that the CSV-based CTC enumeration method, compared to CellSearch, exhibits higher sensitivity and specificity, particularly in detecting CTCs in castration-resistant patients, indicating its potential prognostic value for monitoring prostate cancer progression [33]. Our earlier research focused on detecting programmed death-ligand 1 (PD-L1) positive CTCs in GC using the CSV-enrichment method, but did not compare this to traditional EpCAM detection nor investigate the CSV regulation mechanism and its therapeutic implications [34]. Consequently, our current study addresses these gaps by comparing these detection methods and assessing the predictive value of CSV^+^CTCs for disease progression and prognosis in advanced GC patients, as well as exploring their clinical applications.

In this study, a new high-sensitivity protocol was successfully developed for identifying CTCs in the blood samples of GC patients using the CSV-specific antibody 84-1. Furthermore, it was observed that the markers of both epithelial and EMT phenotypes were expressed in CSV^+^CTCs. Although most of these cells had undergone an EMT transition, a subset still retained epithelial markers such as EpCAM. Due to this heterogeneity, these cells were classified as exhibiting an intermediate EMT phenotype. Thus, employing both CSV and EpCAM markers for CTC enrichment is highly recommended. Additionally, this study further investigated Pritumumab, a human monoclonal antibody targeting glioma cells, and its unique ability to bind to CSV on various cancer cells [35, 36]. This interaction leads to tumor cell death, a novel approach in cancer therapy. Although Pritumumab has been clinically trialed, its effectiveness against GC is less explored. Our findings showed that Pritumumab effectively suppressed GC metastasis in vivo, indicating CSV’s potential as both a marker for CTCs and a therapeutic target in advanced GC.

Building on the understanding of CSV as a crucial marker and therapeutic target, the role of vimentin, another key player in the tumor microenvironment, becomes particularly significant in the context of metastasis and cancer progression. Vimentin, an intermediate filament protein, plays key roles in normal cellular processes, including maintaining cytoskeletal integrity, facilitating cell migration, anchoring the nucleus, and organizing organelles [37]. The upregulated expression of vimentin is associated with cancer metastasis, which involves the dissemination of cancer cells to distant locations in the body [10]. Vimentin promotes metastasis by contributing to EMT, enhancing cancer cell migration and invasion, increasing resistance to apoptosis, and aiding in the colonization of distant organs [10]. Vimentin is present in multiple cellular locations, including the cytoplasm and the cell surface. The cell surface variant, known as cell-surface vimentin (CSV), plays a pivotal role in tumor metastasis. Although free vimentin in the tumor microenvironment can bind to the surfaces of cancer cells and activate the Wnt signaling pathway, enhancing cellular invasion, the mechanisms underlying vimentin’s translocation to the cell surface remain unclear [14]. Our study reveals that the translocation of vimentin from the cytoplasm to the cell membrane is primarily driven by the activation of the IGF-I/IGF-IR pathway, resulting in the phosphorylation of vimentin at serine 39 (S39) for the first time. This marks the first identification of the factors initiating vimentin phosphorylation and its subsequent transport to the cell surface.

Given the critical role of vimentin in cancer metastasis and the emerging understanding of its regulation at the cellular level, exploring the molecular mechanisms behind these processes, such as RNA modifications, becomes imperative for developing more effective cancer therapies. This leads us to investigate the association between RNA modification, particularly m^6^A modification, and its impact on GC metastasis and CSV expression, unveiling new avenues for targeting cancer progression. Our study reveals that FTO is involved in GC metastasis by enhancing CSV expression and promoting CSV^+^CTC formation. FTO is an RNA demethylase involved in the removal of m^6^A modifications from RNA molecules [21]. FTO, implicated in various cancer types, including GC, might influence the m^6^A modification status of transcripts related to vimentin expression, affecting their stability and translation [22, 23]. Similarly, YTHDF2, an m^6^A reader protein, could potentially target vimentin mRNA for degradation, influencing vimentin transcript abundance. Here in our study, in vivo experiments suggest FTO’s role as an upstream regulator of CSV. FTO achieves this by suppressing IGF-IR mRNA decay through m^6^A methylation and m^6^A reader activity, facilitating vimentin translocation. Molecular docking highlights FTO’s interaction with IGF-IR, supported by strong binding affinity with FB23-2. Inhibiting FTO with FB23-2 disrupts the IGF-IR pathway, potentially affecting downstream events, including CSV expression and GC progression. The specificity of FB23-2 towards FTO-IGF-IR axis residues is reinforced by unstable molecular interaction with SER1009. These findings imply FTO’s involvement in regulating CSV^+^CTC formation, offering insights into therapeutic approaches for suppressing GC metastasis.

Nevertheless, this study has some limitations that must be acknowledged. Firstly, being a pilot study, CTCs were assessed at only one blood sampling time point. Secondly, the study enrolled a relatively small number of patients, potentially introducing bias to the results. Thirdly, the median follow-up time of 1.5 years, although typical for a GC study, suggests that data on DFS and PFS should be interpreted with caution. To comprehensively understand the potential of CSV^+^CTCs as a predictor of recurrence, response, and long-term survival of GC patients, it is necessary to conduct long-term follow-up and collect blood samples at different time points.

**Fig. 9:**
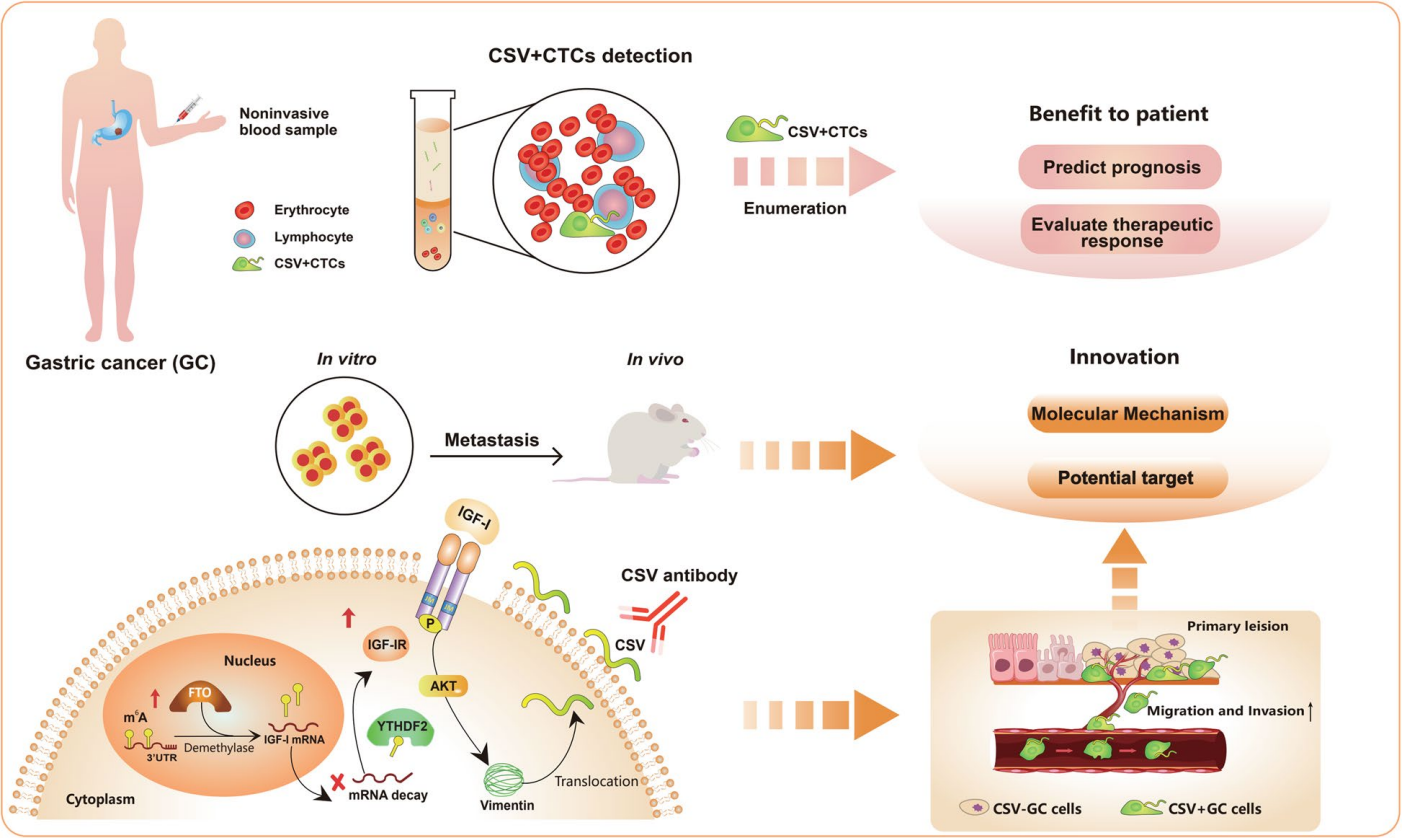
Graphical summary of this study

In conclusion, our study highlights the potential therapeutic implications of inhibiting CSV in advanced GC patients, offering a promising strategy for enhancing therapeutic efficacy and early relapse detection (Fig. 9). However, the transition from laboratory findings to clinical applications poses both feasibility and challenges. Key feasibility considerations include target specificity, delivery systems, and combination therapies, while significant challenges encompass resistance, conducting clinical trials, patient stratification, managing toxicity, and obtaining regulatory approvals. To fully validate the clinical benefits of CTCs in predicting survival, relapse, and therapeutic efficacy, a comprehensive, long-term prospective study is warranted. Notably, interdisciplinary collaboration and sustained research efforts will be vital for realizing the clinical benefits of CSV inhibition in GC therapy.

### Supplementary Information

**Additional file 1: Fig. S1: Representative images of HER-2 IHC staining from the same case (× 100). Fig. S2:The CSV**^**+**^ **GC cells in subcutaneous xenograft mouse models.** BGC-823 cells (4 × 10^6^/mouse) were injected subcutaneously into the right scapula of the nude mice. **(A)** The tumor volume. **(B)** The metastasis nodules were not observed in the lung. **(C)** Flow cytometry showing the number of CSV^+^ GC cells both in the tumor tissues and blood samples. NS = not significant. **Fig. S3: m**^**6**^**A methylation patterns based on a cohort of 300 GC patients from the Asian Cancer Research Group (ACRG). (A)** Clustered heatmap of the gene expression levels of two Eraser genes (FTO and ALKBH5) across samples, with the color bar indicating clinical information distribution based on the clustering results. **(B)** Distribution bar graph of ACRG subtype in EraserCluster A and EraserCluster B. **(C)** Survival prognostic KM curve for EraserCluster A and EraserCluster B groups, with blue and red lines indicating samples in EraserCluster A and EraserCluster B, respectively. **(D)** PCA plot of samples based on two Eraser genes, with blue and red dots representing the samples in EraserCluster A and EraserCluster B, respectively. **(E)** Distribution differences in Enrichment-Score among angiogenesis, CD8 T effector, EMT1, EMT2, EMT3, and Pan-fibroblast TGFβ between EraserCluster A and EraserCluster B. *P < 0.05; *** *P* < 0.001. **Fig. S4: Both proliferation and migration abilities are inhibited in GC cells after FTO inhibitor FB23-2 treatment. (A & B).** The colony-formation capacity of GC cells was evaluated after treatment with FB23-2 at concentrations of 0 μM, 1 μM, and 5 μM). **(C & D)** The migration ability of GC cells was assessed under the same FB23-2 treatment conditions. *** *P* < 0.001. **Fig. S5: Differentially expressed genes in FTO knockdown HGC-27 cells based on RNA-seq analysis.** Different gene profiles after FTO knockdown were presented as Heatmap **(A)** and RNA-seq volcano plot **(B)**. **Fig. S6: Evaluation the role of FTO among cancers and the association of FTO level and IGF-IR expression. (A)** FTO expression in 61 cancers. **(B-Q)** High FTO level is correlated with poor prognosis **(B-G)** and upregulated expression of IGF-IR in cancers **(H-Q)**. **Fig. S7: Knockdown of m**^**6**^**A readers YTHDF1/2/3 in GC cell lines.** Effect of YTHDF1/2/3 knockdown, as verified at mRNA level in both BGC-823 **(A-D)** and HGC-27 cells **(E-H)**. * *P* < 0.05; ** *P* < 0.01; *** *P* < 0.001; NS = not significant. **Fig. S8**: **GSEA showing positive enrichment of EMT process with IGF-I/IGF-IR signaling pathway-related gene sets. (A & B)** GSEA was performed using the JAVA program (https://www.broadinstitute.org/gsea) to identify the IGF-I-related gene sets via MSigDB. **Fig. S9**: **IGF-I is a crucial regulatory factor in GC cells during the EMT process. (A)** GC cells were serum-starved overnight and then treated with or without 100 ng/mL IGF-I for 48 h. Images were captured at 20× magnification. **(B)** Results from migration assays performed using Transwell chamber methods. **(C)** Western blot analysis of cell lysates for EMT markers. **Fig. S10:** TMT quantitative proteomics of HGC-27 cell line after IGF-I stimulation. **(A)** GC cells were serum-starved overnight and then treated with or without 100 ng/mL IGF-I for 48 h. Proteins were extracted for proteomics analysis. Heatmap showing the abundance of EMT genes in the control and IGF-I stimulation groups. **(B & C)** Wheel plot showing the differentially expressed gene enrichment using GO and KEGG. **(D)** PPI subnetwork of differentially expressed EMT-related proteins. **Fig. S11: IGF-I induced the phosphorylation of vimentin in GC cells. (A)** GC cells were serum-starved, followed by treatment with 100 ng/mL IGF-I for 6, 24, or 72 hours. Phosphorylated vimentin levels (at serine residues S39, S72, S82) were measured by Western blot. **(B)** GC cells were serum-starved and subsequently treated with 100 ng/mL IGF-I, both alone and in combination with GSK 1838705A, for 6, 24, or 72 hours. Phosphorylated vimentin levels (S39) were assessed using Western blot assay. **(C)** GC cells were transiently transfected with wild type or specific S39 mutant plasmid for 48 h followed by IGF-I stimulation for 48 h, the expression levels of CSV were detected through flow cytometry. ***P < 0.001; NS = not significant. **Fig. S12**: (A) IHC staining of Ki67 expression of tumor lesions in different groups. Bar = 2mm. (B) Representative H&E-stained sections of metastatic nodules in lung tissue from the mouse model. Bar = 20 μm.

## Data Availability

All the data supporting the conclusions in this article are presented in this article and its additional files.

## Abbreviations

CTCs: Circulating tumor cells
GC: Gastric cancer
EMT: Epithelial-mesenchymal transition
CSV: Cell surface vimentin
IGF-I: Insulin-like growth factor-I
IGF-IR: Insulin-like growth factor-I receptor
RECIST: Response Evaluation Criteria in Solid Tumors
PBMC: Peripheral blood mononuclear cell
FBS: Fetal bovine serum
IHC: Immunohistochemistry
FN1: Fibronectin
HER2: Human epidermal growth factor receptor-2
GSEA: Microarray and genome set enrichment analysis
AUC-ROC: Area under the receiver operating characteristics curve
WES: Whole exome sequencing
PD: Progression disease
SD: Stable disease
PFS: Progression-free survival
OS: Overall survival
DFS: Disease-free survival
TMT: Tandem Mass Tag
MeRIP–qPCR: m^6^A RNA immunoprecipitation–qPCR
SELECT: Single-base elongation- and ligation-based qPCR amplification method
RNA-seq: RNA sequencing
FTO: Fat mass and obesity associated protein
VIM: Vimentin
