# Supplementary material for: Exploring new frontiers: cell surface vimentin as an emerging marker for circulating tumor cells and a promising therapeutic target in advanced gastric Cancer
Source: J Exp Clin Cancer Res. 2024 Apr 30;43:129. doi: 10.1186/s13046-024-03043-6 (PMC11059585; doi:10.1186/s13046-024-03043-6)
Supplement: Supplementary file 1 — Additional file 1: Fig. S1: Representative images of HER-2 IHC staining from the same case (× 100). Fig. S2:The CSV+ GC cells in subcutaneous xenograft mouse models. BGC-823 cells (4 × 10^6^/mouse) were injected subcutaneously into the right scapula of the nude mice. (A) The tumor volume. (B) The metastasis nodules were not observed in the lung. (C) Flow cytometry showing the number of CSV+ GC cells both in the tumor tissues and blood samples. NS = not significant. Fig. S3: m6A methylation patterns based on a cohort of 300 GC patients from the Asian Cancer Research Group (ACRG). (A) Clustered heatmap of the gene expression levels of two Eraser genes (FTO and ALKBH5) across samples, with the color bar indicating clinical information distribution based on the clustering results. (B) Distribution bar graph of ACRG subtype in EraserCluster A and EraserCluster B. (C) Survival prognostic KM curve for EraserCluster A and EraserCluster B groups, with blue and red lines indicating samples in EraserCluster A and EraserCluster B, respectively. (D) PCA plot of samples based on two Eraser genes, with blue and red dots representing the samples in EraserCluster A and EraserCluster B, respectively. (E) Distribution differences in Enrichment-Score among angiogenesis, CD8 T effector, EMT1, EMT2, EMT3, and Pan-fibroblast TGFβ between EraserCluster A and EraserCluster B. *P < 0.05; *** P < 0.001. Fig. S4: Both proliferation and migration abilities are inhibited in GC cells after FTO inhibitor FB23-2 treatment. (A & B). The colony-formation capacity of GC cells was evaluated after treatment with FB23-2 at concentrations of 0 μM, 1 μM, and 5 μM). (C & D) The migration ability of GC cells was assessed under the same FB23-2 treatment conditions. *** P < 0.001. Fig. S5: Differentially expressed genes in FTO knockdown HGC-27 cells based on RNA-seq analysis. Different gene profiles after FTO knockdown were presented as Heatmap (A) and RNA-seq volcano plot (B). Fig. S6: Evaluation the role of FT [file 13046_2024_3043_MOESM1_ESM.docx]

**Supplementary Text**

**Material and methods**

**Immunofluorescent imaging and analysis**

For immunofluorescent imaging, the selected cell pellet was mixed with MACS buffer (Miltenyi Biotec) and stained with the 84–1 antibody in a 15 mL tube at room temperature (RT) for 1 hour. Cells were cytospun onto Polylysine^TM^ microscope adhesion slides (Thermo Fisher Scientific) using CytoFuge (Iris). Later, those cells were fixed by 4% paraformaldehyde (Fisher Scientific) for 10 minutes followed by blocking in blocking buffer (1% FBS in PBS) for 1 hour. For the staining of other specific markers, such as CD45, Cytokeratin, N-cadherin, EpCAM, E-cadherin (Cell Signaling Technology), the selected cells were incubated with primary antibody overnight at 4 degrees after permeabilization with 0.5% Triton X-100 for 15 mins. On the second day, the slides were washed with PBS three times and stained with Alexa Fluor-488 for 84-1, Alexa Fluor-555 for other markers, and DAPI for nuclei staining for 1 h at RT in dark room. Images were captured and analyzed under the 100×oil objective with the confocal acquisition software FV10-ASW 3.0 (Olympus, Tokyo, Japan).

**Spiking assay**

To demonstrate the sensitivity of CTCs captured by the 84-1 antibody, 0, 5 and 10 BGC-823 gastric cancer cells labeled with CFSE tracking dye (Invitrogen) were spiked into 1×10^6^ PBMCs (nearly 6 mL of whole blood isolated from healthy donor). All cells used for spiking assay were subjected to 84-1^+^ selection before spiking analysis. Spiking experiments were performed in triplicate to ensure the reproducibility of the cell recovery rate using this method.

**Cell culture**

Human gastric cancer cell lines were kindly provided by Dr. Yunpeng Liu (China Medical University). All the GC cells were cultured in RPMI-1640 medium (Gibco) supplemented with 10% FBS and penicillin (10 U/mL) and streptomycin (100 mg/mL) in an atmosphere of 5% CO2 at 37 degrees. Cells were sub-cultured every 2-3 days and harvested in their logarithmic phase of growth. Live cells with viability greater than 98% were used for experiments.

**Flow cytometry**

A total of 5×10^5^ cells was detached with a non-enzymatic dissociation buffer and stained with the 84-1 monoclonal antibody (1:100); mouse primary antibody (CST) was used as an isotype control. Later, cells were rinsed twice in PBS and labeled for secondary antibody using Alexa Fluor-488 (CST). Cells were then washed twice in PBS and used for data acquisition immediately using Attune flow cytometer (Thermo Fisher Scientific). Fifty thousand cells were counted for flow analysis. The data were analyzed using FlowJo software (Treestar).

**Western Blot Assay**

Total protein was extracted using the radioimmunoprecipitation assay (RIPA) lysis buffer (Beyotime, China) according to the manufacturer’s instructions. Membrane and cytoplasmic protein fractions of GC cells were obtained with Mem-PER^TM^ Plus membrane protein extraction kit (Thermo Fisher Scientific). All the samples were eluted by boiling water at 100 degrees for 5 mins with 3×sampling buffer. Equal amounts of protein samples (10 ug) were separated by 10% SDS-PAGE and electronically transferred to PVDF membranes (Millipore, USA). The blots were incubated with primary antibodies at 4 degrees overnight after blocking with 5% skim milk in 1× TBST. Later, the blots were incubated with secondary antibodies (Cell Signaling Technology) for 30 mins at RT on the next day. After washing with 1× TBST, proteins were visualized by ECL reagent (Cell Signaling Technology).

**Migration Assay**

Migration assay was performed using Transwell chambers (Corning) with 8-um pore size membranes. 1 × 10^4^ cells/well were seeded into the upper chamber with 200 μL serum-free RPMI 1640 medium. Then the upper chamber was inserted into 24-well culture dish with 500 μL of RPMI 1640 containing 2.5% FBS. After incubation for 24 hours, the culture media in the upper chamber and non-migrated cells on the inner side were carefully removed with a cotton swab. The migrated cells on the lower side were stained with crystal violet for 2 hours and counted in five different fields at × 10 magnification under the microscope.

***Lentivirus transfection***

*To genetically suppress phosphorylation of vimentin, the S39 phosphorylation VIM mutant (S39A) in which S39 was replaced with alanine was generated from the GV492 vector (GeneChem, Shanghai, China) containing Ubi-MCS-3FLAG-CBh gcGFP-IRES-puromycin. The gastric cancer cell BGC-823 were transfected following the manufacturer’s instructions (Gene Chem, Shanghai, China).*

**In vivo treatments**

Nude mice weighed 18-20g (male) were obtained from Shanghai SLAC laboratory animal co., Ltd. All animals are raised in the SPF barrier system of Shanghai University of Traditional Chinese Medicine. All animal experiments were approved by the Institutional Animal Care and Use Committee of Shanghai University of Traditional Chinese Medicine. After one-week acclimation, mice were injected with 5×10^6^/100ul 823-luc cells through tail vein to establish lung metastasis mouse model. In vivo image was taken on day 4 and day 14 using IVIS Lumina XR Imaging System to monitor lung metastasis in mice. On the 4^th^ day after injection when lung metastasis was confirmed, model mice were randomly divided into 4 groups: control group, Pritumumab group, GSK1838705A group and Pritumumab + GSK1838705A group, daily treated with 2mg/kg Pritumumab (intraperitoneally), 4mg/kg GSK1838705A (intragastrically), or combined, respectively for 2 weeks. For the treatment of FB23-2 when applicated, model mice were randomly divided into 2 groups: control group and FB23-2 group, daily treated with DMSO vehicle or 4mg/kg FB23-2 (intraperitoneally), respectively for 2 weeks.

**Tissue processing and flow cytometry**

After anesthetized with 1 % sodium pentobarbital, whole blood of mice was taken from the eyeball followed by a 7 minute’s’ red blood cell lysis. Blood cell suspensions were resuspended with PBS prior to cell surface staining with an appropriate amount of FITC-CSV antibody (Abnova). Data were acquired using CytoFlex S Flow Cytometer (Beckman Coulter).

**H&E Staining**

Tumors were taken and immersed in 4% paraformaldehyde. The sections were then dehydrated by ethanol gradient, transparent in xylene, and embedded in paraffin for staining. The sections stained with hematoxylin and eosin were observed under a microscope.

**Immunohistochemistry (IHC)**

Tumors were taken and immersed in 4% paraformaldehyde. The sections were then dehydrated by ethanol gradient, transparent in xylene, and embedded in paraffin followed by incubating with 3% hydrogen peroxide. Antigen retrieval was achieved by microwaving the sections in 0.01 mol/L citrate buffer. The immunostaining was undertaken with antibodies against HER2, E-cadherin, Vimentin, Fibronectin (FN1), p-AKT, AKT, NF-κB, respectively (Cell Signaling Technology), followed by incubation with secondary antibody. The section was finally stained with hematoxylin and observed under a microscope.

**Microarray and Genome Set Enrichment Analysis (GSEA)**

Based on the mRNA profile of the 20530 genes downloaded from UCSC database, GSEA was performed by the JAVA program (https://www.broadinstitute.org/gsea) to identify the IGF-I‐related gene sets using MSigDB H: hallmark gene sets as functional gene sets. After performing 1000 permutations, the gene sets with FDR q < 0.25 and P < 0.05 were significantly enriched. The gene‐set network and the corresponding heat map were then constructed with R igraph and R heatmap package. Relationships between functional terms were visualized in Cytoscape [version 2.8.3] (PMID: 21149340) with the Enrichment Map package [version 1.2] (PMID: 21085593). All microarray and GSEA analysis were performed on a node running Debian Linux [version 6.0.5].

**DNA collection and whole-exome sequencing**

DNA was extracted from two primary GC tissue samples and paired CTCs cell population enriched from patients’ peripheral blood samples. DNA concentration was measured by Qubit® DNA Assay Kit in Qubit® 2.0 Flurometer (Invitrogen, USA). For library preparation, sequencing libraries were generated using Agilent SureSelect Human All Exon V6 kit (Agilent Technologies, CA, USA) based on manufacturer’s recommendations. The DNA libraries were sequenced on Illumina Hiseq platform followed by cluster generation.

**TMT quantitative proteomics**

All the proteins were extracted and digested with trypsin followed by labelling with 10-plex TMT reagents. Then all labeling samples were mixed with equal volume, desalted, and lyophilized. TMT-labeled peptide mix was fractionated using a C18 column (Waters BEH C18, 4.6×250 mm, 5 μm) on a Rigol L3000 HPLC. Collected fractions were concatenated into 10 fractions. The TMT-labeled proteome was analyzed using Q Exactive^TM^ HF-X mass spectrometer (Thermo Fisher). Data analysis and visualization of TMT data were conducted by Novogene Bioinformatics Technology Co., Ltd (Beijing, China) using the Proteome Discoverer (PD 2.2, Thermo Fisher Scientific) platform and R statistical framework. For data analysis, the proteins whose quantitation significantly different between IGF-I treatment and control groups in GC cell lines (p < 0.05 and FC > *1.5 or FC < * 1.2 [fold change, FC]) were defined as differentially expressed proteins.

**Differential expression analysis and enrichment analysis**

Protein differential expression and enrichment was searched for bioinformatic analysis. Proteins with a p-value <0.05 and |log2FC| >1 were defined as to be significantly different for further analysis. The *clusterprofiler* R package was performed on the differentially expressed proteins after IGF-I treatment in GC cells in order to explore the potential molecular functions based on GO and KEGG pathway enrichment analysis. Gene set enrichment analysis (GSEA) was performed via the molecular signatures database MSigDB by the GSEA software v. 3.0.

**RNA sequencing (RNA-Seq) and m^6^A sequencing (m^6^A-Seq)**

The samples are control and knockdown cells. Total RNA was extracted using TRIzol^TM^ Reagent. A total amount of 1 µg RNA per sample was used as input material for the RNA sample preparations. Sequencing libraries were generated using NEBNext® UltraTM RNA Library Prep Kit for Illumina® (NEB, USA) following manufacturer’s recommendations and index codes were added to attribute sequences to each sample. The MeRIP library was prepared by smart-seq method. The concentration of total RNA was measured by Qubit RNA HS assay kit. Both the input samples without IP and the m6A IP samples were subjected to 150-bp, paired-end sequencing on an Illumina NovaSeq 6000 sequencer. The RNA seq and MeRIP-seq were sent to a laboratory, Genechem (Shanghai Genechem Co., LTD) for RNA sequencing.

**siRNA transfection**

HGC-27 cells and BGC-823 cells were transfected with siRNA (JTS Scientific, China), targeting negative control (NC), FTO, YTHDF1, YTHDF2 and YTHDF3 using the riboFECT™ CP kit (RIBOBIO, Guangzhou, China) according to the manufacturer’s instructions.

**Quantitative real-time PCR (qPCR)**

Total RNAs from the transfected GC cells were extracted with EZB-press RNA Purification Kit (EZBioscience, CA, USA). qPCR assays were carried out using a QuantStudio™ 3 Real-Time PCR System (Thermo Fisher Scientific, CA, USA) with 4×Reverse Transcription Master Mix (EZBioscience, CA, USA) and 2×SYBR Green qPCR Master Mix (ROX1 plus) (EZBioscience, CA, USA). The threshold cycle number (CQ) was analyzed in triplicate for each sample. The CQ values for FTO, IGF-I, IGF-IR, YTHDF1, YTHDF2, YTHDF3, CXADR, IGFBP5 and GAPDH. GAPDH (Sango Biotech, Shanghai, China) was used for normalization. The primer sequences used for qRT-PCR are shown in Supplementary Table1.

**mRNA stability assay**

To measure RNA stability in GC cells after FTO knockdown, actinomycin D (Act-D, Sigma, USA) at 5 μg/ml was added to GC cells. After incubation at the indicated times, total RNA was isolated by EZB-press RNA Purification Kit (EZBioscience, CA, USA). qPCR was conducted to quantify the relative levels of target mRNA and GAPDH was used for normalization.

**m^6^A RNA immunoprecipitation (MeRIP)**

MeRIP-qPCR was performed using BersinBio^TM^ MeRIP kit (BersinBio, Guangzhou, China) according to the manufacturer’s instructions. Briefly, the extracted total RNA was sheared into fragments with approximately 300 bp. Then, RNA samples were immunoprecipitated with magnetic beads pre-coated with either anti-m^6^A antibody (Abcam) or anti-IgG antibody (BersinBio). Next, the m^6^A-modified RNA fragments were washed and harvested according to the instruction manual. qPCR was performed to measure the methylated RNA expression levels. The specific primers for MeRIP-qPCR are shown in Supplementary Table 1.

**FTO-assisted SELECT method**

We used the Epi-SELECT^TM^ m^6^A site identification kit (Epibiotek) to measure m^6^A levels in GC cells after FTO inhibition. Data analysis and visualization were conducted by Guangzhou Epibiotek Co., Ltd (Guangzhou, China). This novel method uses the SELECT qPCR technique to detect m^6^A modification sites, and FTO demethylation is used to identify these sites further. The kit involves annealing and extending probes on either side of m^6^A sites, which are then connected using the SELECT^TM^ connection enzyme. The SELECT^TM^ DNA polymerase is used for efficient single-base extension, and products are then subjected to qPCR detection after heating. By comparing the difference in CT values after FTO demethylation treatment, products formed from RNA templates without m^6^A modification sites were practically identical. However, there was a significant increase in CT values of products formed from RNA templates with m^6^A modifications, indicating successful detection and identification using qPCR analysis.

**Molecular docking and Molecular dynamics simulation**

We obtained the IGF-IR structure from RCSB Protein Data Bank and prepared it using the Protein Preparation Wizard in Schrödinger. Ligand was pre-treated with LigPrep using OPLS_4 force field. Protein Contacts Atlas was utilized to analyze non-covalent interactions of the IGF-IR complex. After this, Molecular docking was used to obtain docking score and 2D workspace that allow analysis of ligand-receptor interactions. To evaluate structural changes and stability, Molecular Dynamics (MD) simulation was performed using the Desmond module with SPC as the solvent model, then optimization and minimization were performed by OPLS_4 force field. Finally, MD simulation for 100 ns at 1.013 bar and 300 K resulting in protein-ligand RMSD and protein RMSF.

**Statistical analysis**

Data reported here are expressed as means standard deviation. Statistical analyses were performed using the Prism software program (GraphPad Software). Differences in baseline characteristics and CTC counts among patients with pre- and post-surgery, unresectable and those with metastatic GC were analyzed using the Fisher exact test and t-tests. Diagnostic performance of CTC counts using either of EpCAM or CSV methods was evaluated by a composite area under the receiver operating characteristics curve (AUC-ROC) using SPSS 20.0 and R package. P values less than 0.05 were considered signiﬁcant.

**Supplementary Tables**

**Table S1 Primer sequences for qPCR**

| **Gene name** | **Forward** | **Reverse** |
| --- | --- | --- |
| FTO | ATCTCAATGCCACCCACCAACAC | CTCCATCTTCTTCCACAGTGCTTCC |
| IGF-I | TGTCCTCCTCGCATCTCTTCTACC | CGCAATACATCTCCAGCCTCCTTAG |
| IGF-IR | GGGTCGTTTGGGATGGTCTATGAAG | AGGCTTGGAGGTGCTAGGACTG |
| YTHDF1 | TCAATGAGGCTCCGTGGTCTACTG | GCTGAGGGTGTCGCTGTGAAAG |
| YTHDF2 | CAGACACAGCCATTGCCTCCAC | AAGCAGCATCCAGTCTCTTGTTACC |
| YTHDF3 | ATTGAGCAAGGCATGACTGGACTG | TAGGAGGTGGTGGTACAGCAGAAC |
| IGFBP5 | TGAAGAAGGACCGCAGAAAGAAGC | CTTGTCCACGCACCAGCAGATG |
| CXADR | CTTCAGGTGCGAGATGTTA | AGCTGTATGTCCCAGAGTA |

**Table S2. Statistical and distribution comparison of clinical information samples in EraserCluster.A and EraserCluster.B**

| **Characteristics total cases** | **N of case 300** | **Subtype** | | **P value** |
| --- | --- | --- | --- | --- |
|  |  | **EraserCluster.A (N=144)** | **EraserCluster.B (N=156)** |  |
| ACRG subtype |  |  |  |  |
| MSS/TP53- | 107 | 46 | 61 | 5.61E-09 |
| MSS/TP53+ | 79 | 41 | 38 |  |
| MSI | 68 | 50 | 18 |  |
| EMT | 46 | 7 | 39 |  |
| Stage |  |  |  |  |
| Stage I | 31 | 24 | 7 | 2.83E-03 |
| Stage II | 96 | 48 | 48 |  |
| Stage III | 96 | 41 | 55 |  |
| Stage IV | 77 | 31 | 46 |  |
| Histology |  |  |  |  |
| Diffuse | 135 | 51 | 84 | 5.51E-03 |
| Intestinal | 148 | 83 | 65 |  |
| Mixed | 17 | 10 | 7 |  |
| Age(years) |  |  |  |  |
| ≤65 | 172 | 80 | 92 | 5.61E-01 |
| >60 | 128 | 64 | 64 |  |
| Status |  |  |  |  |
| Alive | 116 | 56 | 60 | 9.99E-01 |
| Dead | 184 | 88 | 96 |  |

**Table S3. EMT-related genes list**

| **EMT-related genes list** | | | | | | |
| --- | --- | --- | --- | --- | --- | --- |
| ABCC3 | ABHD11 | ADGRG1 | AGR2 | AKAP12 | AKR1B10 | AKT3 |
| ANGPTL2 | ANK2 | AP1M2 | AP1S2 | ARHGAP32 | ARHGAP8 | ASPN |
| ATP2C2 | AXL | AZGP1 | BCAS1 | BGN | BICC1 | BIK |
| BNC2 | BSPRY | C1ORF54 | C1R | C1S | C4ORF19 | CALD1 |
| CAV1 | CAVIN1 | CBLC | CCL2 | CCL8 | CD163 | CD24 |
| CD2AP | CDH1 | CDH11 | CDH2 | CDK14 | CDS1 | CEACAM1 |
| CEACAM5 | CEACAM6 | CEACAM7 | CEP170 | CHN1 | CHRDL1 | CKMT1A |
| CLDN3 | CLDN4 | CLDN7 | CLEC2B | CLIC4 | COL14A1 | COL15A1 |
| COL5A2 | COL6A1 | COL6A2 | COLEC12 | CORO2A | CRISPLD2 | CRYAB |
| CSF2RB | CSRP2 | CTSK | CXADR | CXCL12 | CXCL13 | CXCR4 |
| CYB561 | CYP1B1 | DCN | DDR1 | DDR2 | DHCR24 | DPT |
| DPYSL3 | DSE | DSG2 | DSP | ECM2 | EFEMP1 | EFEMP2 |
| EHF | ELF3 | ELMO3 | EMP3 | ENPP2 | EPB41L4B | EPCAM |
| EPN3 | EPS8L1 | EPS8L2 | ERBB2 | ERBB3 | ESRP1 | ESRP2 |
| EVI2A | EXPH5 | EZR | F11R | F13A1 | FA2H | FAM174B |
| FAP | FBLN1 | FBN1 | FERMT2 | FGFR3 | FGL2 | FHL1 |
| FLI1 | FLRT2 | FN1 | FSTL1 | FUT2 | FUT3 | FXYD3 |
| FXYD6 | FYN | GALE | GALNT3 | GALNT7 | GAS1 | GDF15 |
| GEM | GFPT2 | GIMAP4 | GIMAP6 | GLIPR1 | GLYR1 | GMDS |
| GNG11 | GPM6B | GPRC5A | GREM1 | GRHL2 | GUCY1B1 | GZMK |
| HDHD3 | HEG1 | IFFO1 | IGF1R | IGFBP5 | IL10RA | IL20RA |
| INAVA | IRF6 | ISLR | ITM2A | JAM2 | JAM3 | JCAD |
| JUP | KCNJ8 | KRT18 | KRT19 | KRT7 | KRT8 | LAD1 |
| LHFPL6 | LLGL2 | LOX | LRRC1 | LSR | LY96 | MAF |
| MAFB | MANSC1 | MAP1B | MAP7 | MAPK13 | MEOX2 | MFAP4 |
| MISP | MLPH | MMP2 | MOXD1 | MPDZ | MPZL2 | MRC1 |
| MS4A4A | MS4A6A | MST1R | MUC1 | MYH10 | MYL9 | MYLK |
| MYO5C | MYO6 | NAP1L3 | NQO1 | NR3C1 | NUAK1 | OCLN |
| OLFML2B | OLFML3 | OR7E14P | OVOL2 | PALM2AKAP2 | PCOLCE | PDGFC |
| PDZRN3 | PERP | PHLDA2 | PKP3 | PLEKHO1 | PLLP | PLN |
| PLPP2 | PLS1 | PLXNC1 | PMP22 | POF1B | PPL | PRR15L |
| PRSS8 | PSCA | PTGDS | PTGIS | PTK6 | PTPRC | PTPRF |
| PTX3 | QKI | RAB11FIP1 | RAB25 | RAPGEFL1 | RARRES2 | RBM47 |
| RECK | RGS2 | RNF128 | RUNX1T1 | S100A14 | S100P | SACS |
| SAMSN1 | SCNN1A | SDC2 | SDC4 | SEPTIN6 | SERPINF1 | SERPING1 |
| SFRP1 | SFRP4 | SH3YL1 | SLC16A5 | SLC22A18 | SLC2A3 | SLC35A3 |
| SLC44A4 | SLIT2 | SNAI2 | SOBP | SORD | SPAG1 | SPARC |
| SPARCL1 | SPDEF | SPINT1 | SPINT2 | SPOCK1 | SRGN | SRPX |
| ST14 | STAP2 | STON1 | STYK1 | SYNE1 | SYNE3 | SYNGR2 |
| SYNM | SYT11 | TACSTD2 | TAGLN | TCF4 | TFF1 | TFF3 |
| TJP2 | TJP3 | TMC5 | TMEM30B | TMPRSS2 | TMPRSS4 | TNC |
| TNS1 | TOB1 | TOM1L1 | TOX3 | TPD52 | TPM2 | TRPC1 |
| TRPM4 | TSPAN1 | TSPAN8 | TTC39A | TUBA1A | TUBB6 | TUFT1 |
| TWIST1 | UCHL1 | VAMP8 | VAV3 | VCAM1 | VCAN | VIM |
| VSIG4 | WIPF1 | WWTR1 | ZCCHC24 | ZEB1 | ZEB2 | ZFPM2 |

**Supplementary Figures**

**
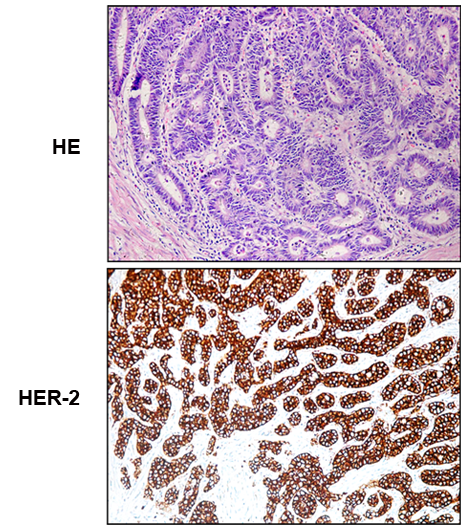
**

**Fig. S1: Representative images of HER-2 IHC staining from the same case (x100).**


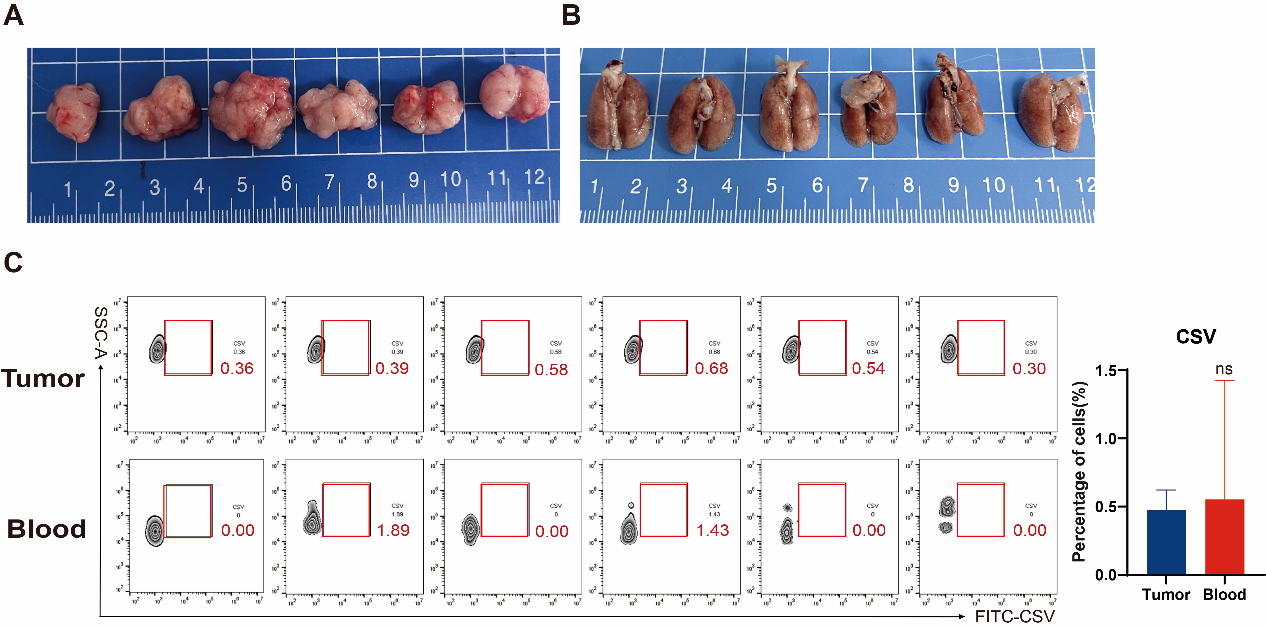


**Fig. S2：The CSV^+^ GC cells in subcutaneous xenograft mouse models.** BGC-823 cells (4×10^6^/mouse) were injected subcutaneously into the right scapula of the nude mice. **(A)** The tumor volume. **(B)** The metastasis nodules were not observed in the lung. **(C)** Flow cytometry showing the number of CSV^+^ GC cells both in the tumor tissues and blood samples. NS = not significant.

**
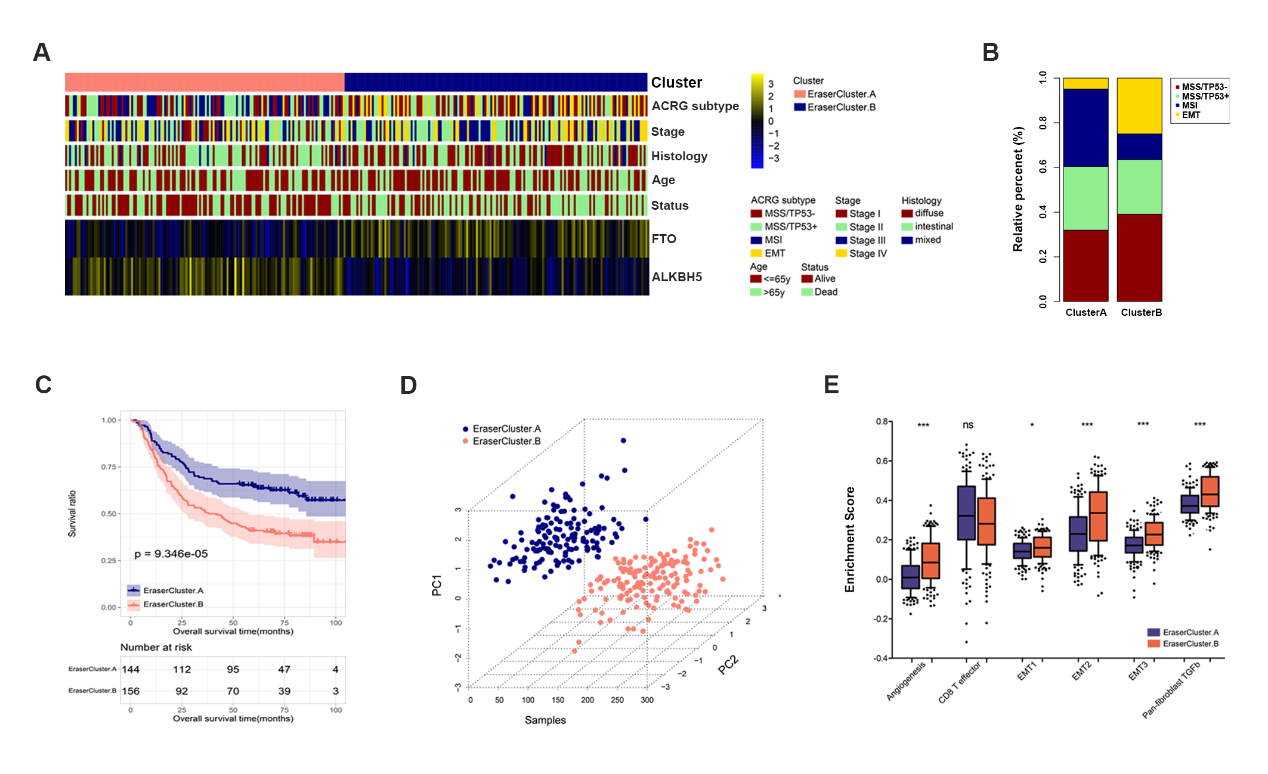
**

**Fig. S3: m^6^A methylation patterns based on a cohort of 300 GC patients from the Asian Cancer Research Group (ACRG).** **(A)** Clustered heatmap of the gene expression levels of two Eraser genes (FTO and ALKBH5) across samples, with the color bar indicating clinical information distribution based on the clustering results. **(B)** Distribution bar graph of ACRG subtype in EraserCluster A and EraserCluster B. **(C)** Survival prognostic KM curve for EraserCluster A and EraserCluster B groups, with blue and red lines indicating samples in EraserCluster A and EraserCluster B, respectively. **(D)** PCA plot of samples based on two Eraser genes, with blue and red dots representing the samples in EraserCluster A and EraserCluster B, respectively. **(E)** Distribution differences in Enrichment-Score among angiogenesis, CD8 T effector, EMT1, EMT2, EMT3 and Pan-fibroblast TGFβ between EraserCluster A and EraserCluster B. **P* < 0.05; ** *P* < 0.01; *** *P* < 0.001.


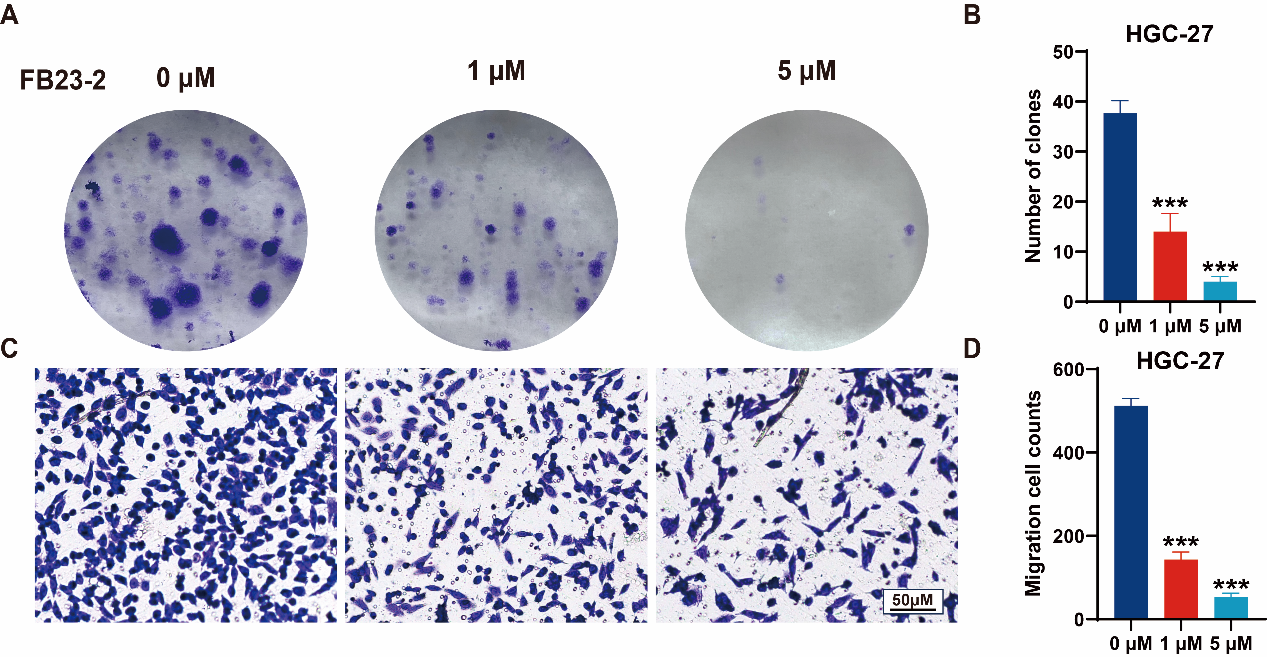


**Fig. S4: Both proliferation and migration abilities are inhibited in GC cells after FTO inhibitor** **FB23-2 treatment. (A & B).** The colony-formation capacity of GC cells was evaluated after treatment with FB23-2 at concentrations of 0μM, 1μM, and 5μM). **(C & D)** The migration ability of GC cells was assessed under the same FB23-2 treatment conditions. *** P < 0.001.

**
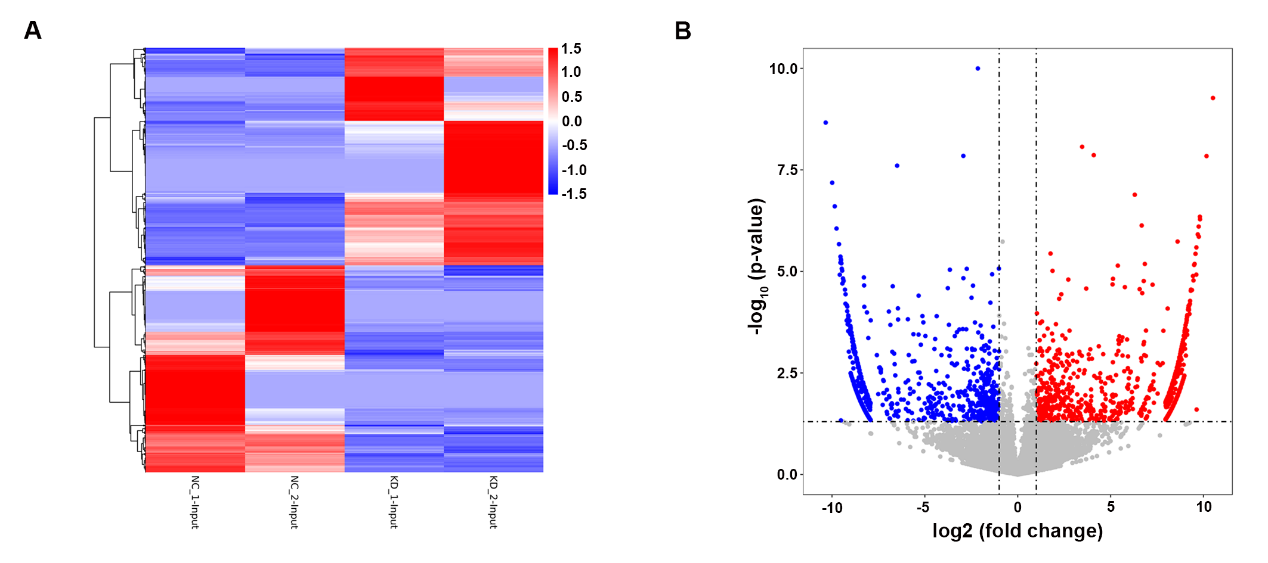
**

**Fig. S5: Differentially expressed genes in FTO knockdown HGC-27 cells based on RNA-seq analysis.** Different gene profiles after FTO knockdown were presented as Heatmap **(A)** and RNA-seq volcano plot **(B)**.

**
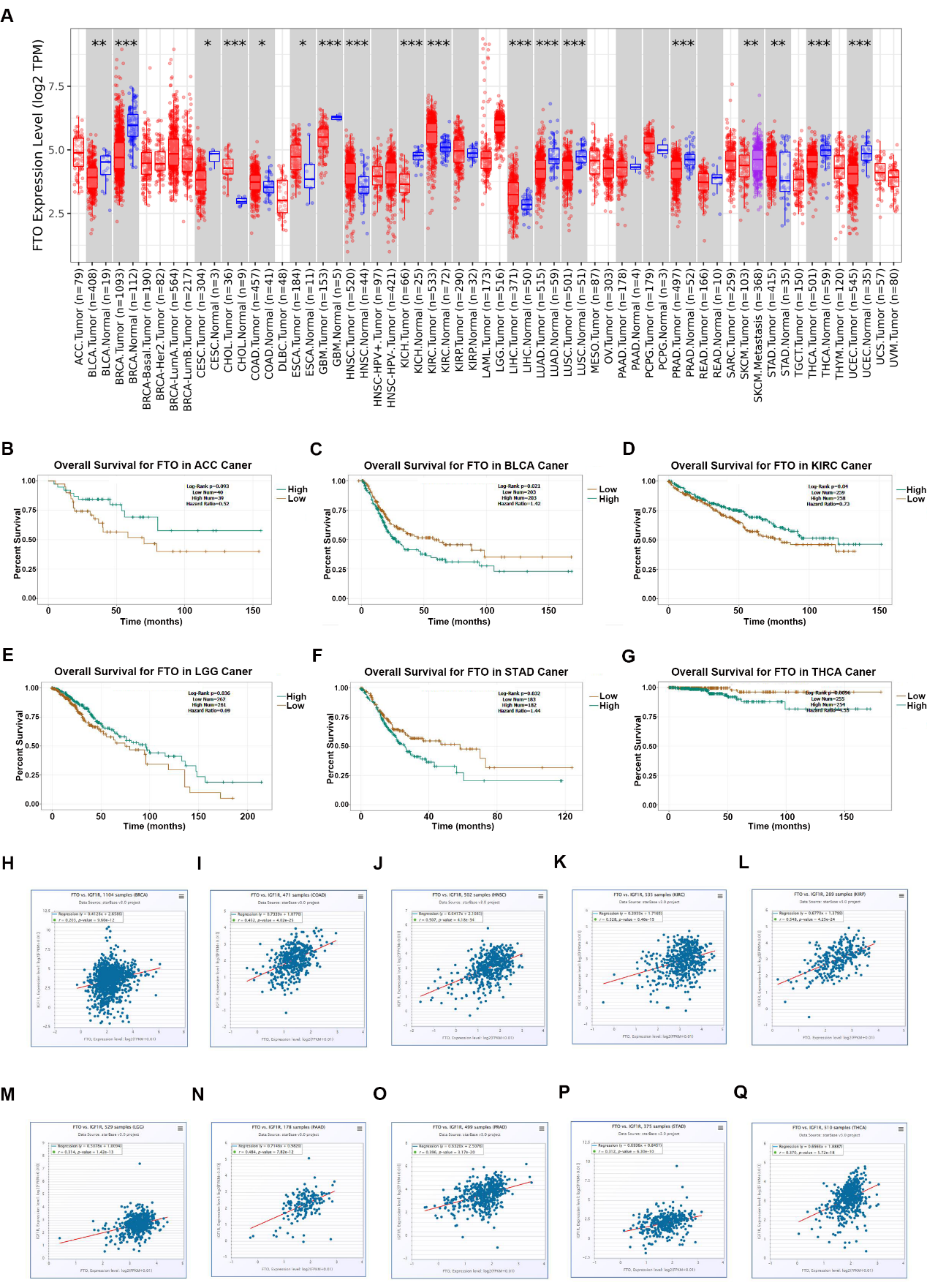
**

**Fig. S6: Evaluation** **the role of FTO among cancers and association of FTO level and IGF-IR expression.** **(A)** FTO expression in 61 cancers. **(B)** High FTO level is correlated with poor prognosis **(B)** and upregulated expression of IGF-IR in cancers **(C)**.

**
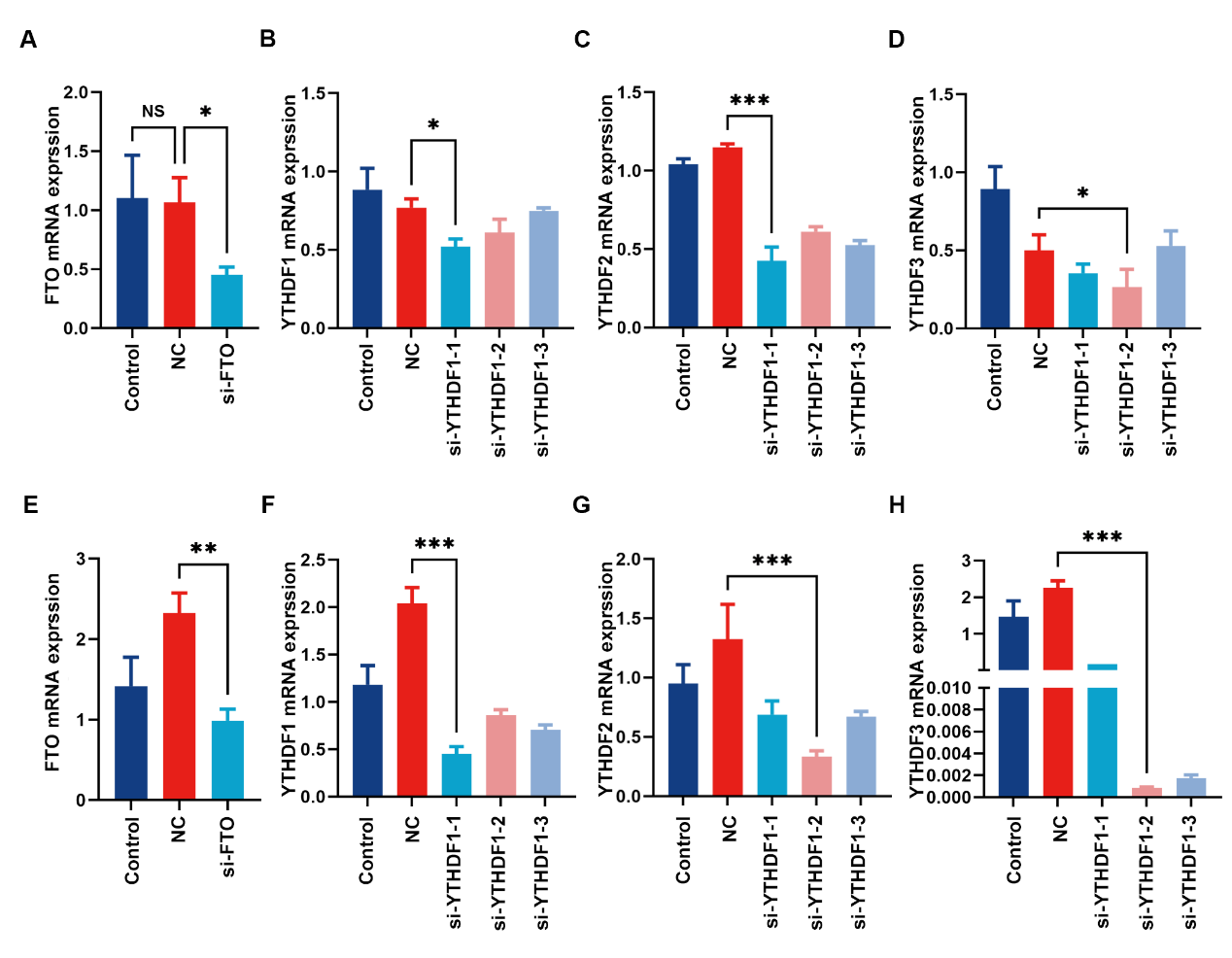
**

**Fig. S7: Knockdown of m^6^A readers** **YTHDF1/2/3 in GC cell lines.** Effect of YTHDF1/2/3 knockdown, as verified at mRNA level in both BGC-823 **(A-D)** and HGC-27 cells **(E-H)**. * *P* < 0.05; ** *P* < 0.01; *** *P* < 0.001; NS = not significant.

**
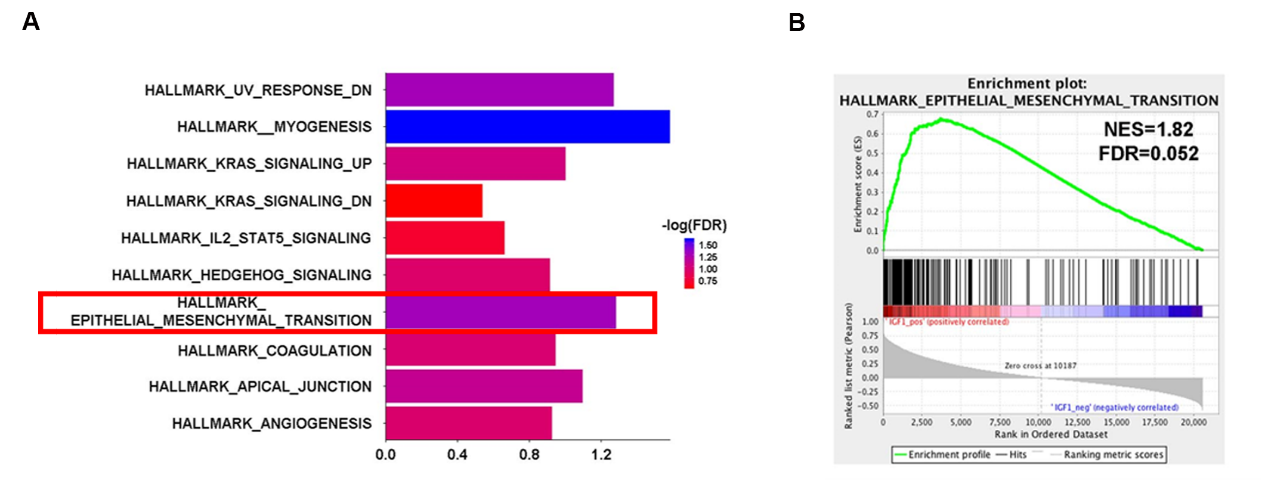
**

**Fig. S8**: **GSEA showing positive enrichment of EMT process with IGF-I/IGF-IR signaling pathway-related gene sets. (A & B)** GSEA was performed using the JAVA program (https://www.broadinstitute.org/gsea) to identify the IGF-I‐related gene sets via MSigDB.

**
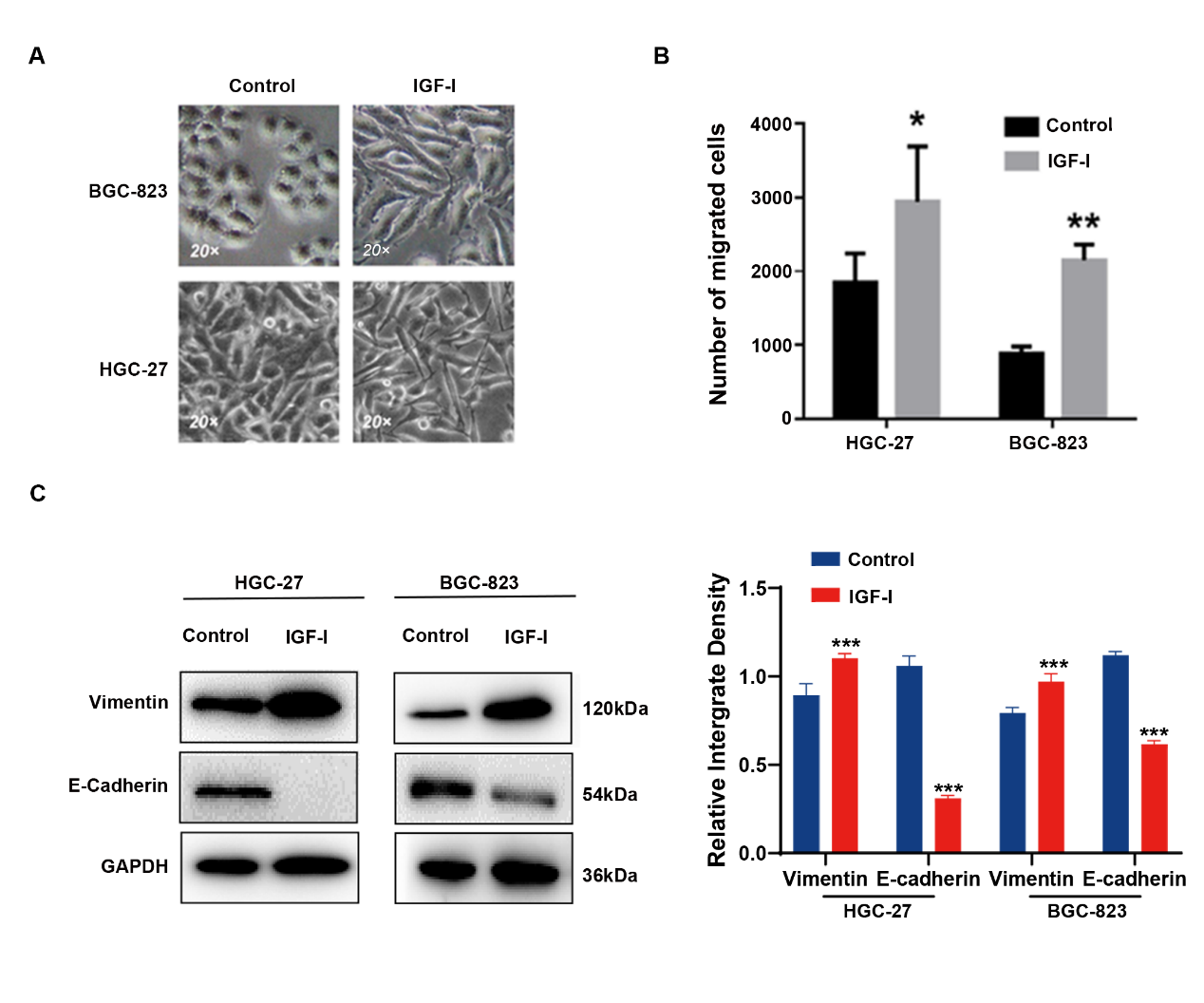
**

**Fig. S9**: **IGF-I is a crucial regulatory factor in GC cells during the EMT process.** **(A)** GC cells were serum-starved overnight and then treated with or without 100 ng/mL IGF-I for 48 h. Images were captured at 20× magnification. **(B)** Results from migration assays performed using Transwell chamber methods. **(C)** Western blot analysis of cell lysates for EMT markers.

**
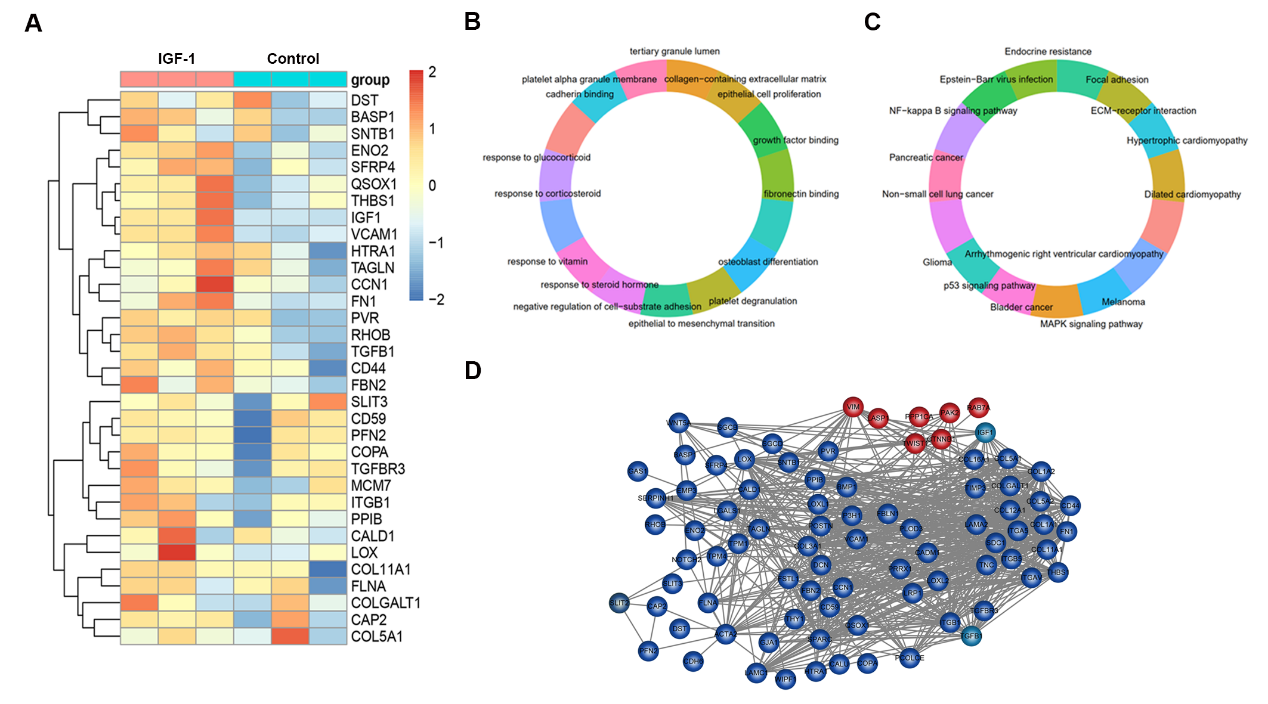
**

**Fig. S10:** TMT quantitative proteomics of HGC-27 cell line after IGF-I stimulation. **(A)** GC cells were serum-starved overnight and then treated with or without 100 ng/mL IGF-I for 48 h. Proteins were extracted for proteomics analysis. Heatmap showing the abundance of EMT genes in the control and IGF-I stimulation groups. **(B & C)** Wheel plot showing the differentially expressed gene enrichment using GO and KEGG. **(D)** PPI subnetwork of differentially expressed EMT-related proteins.


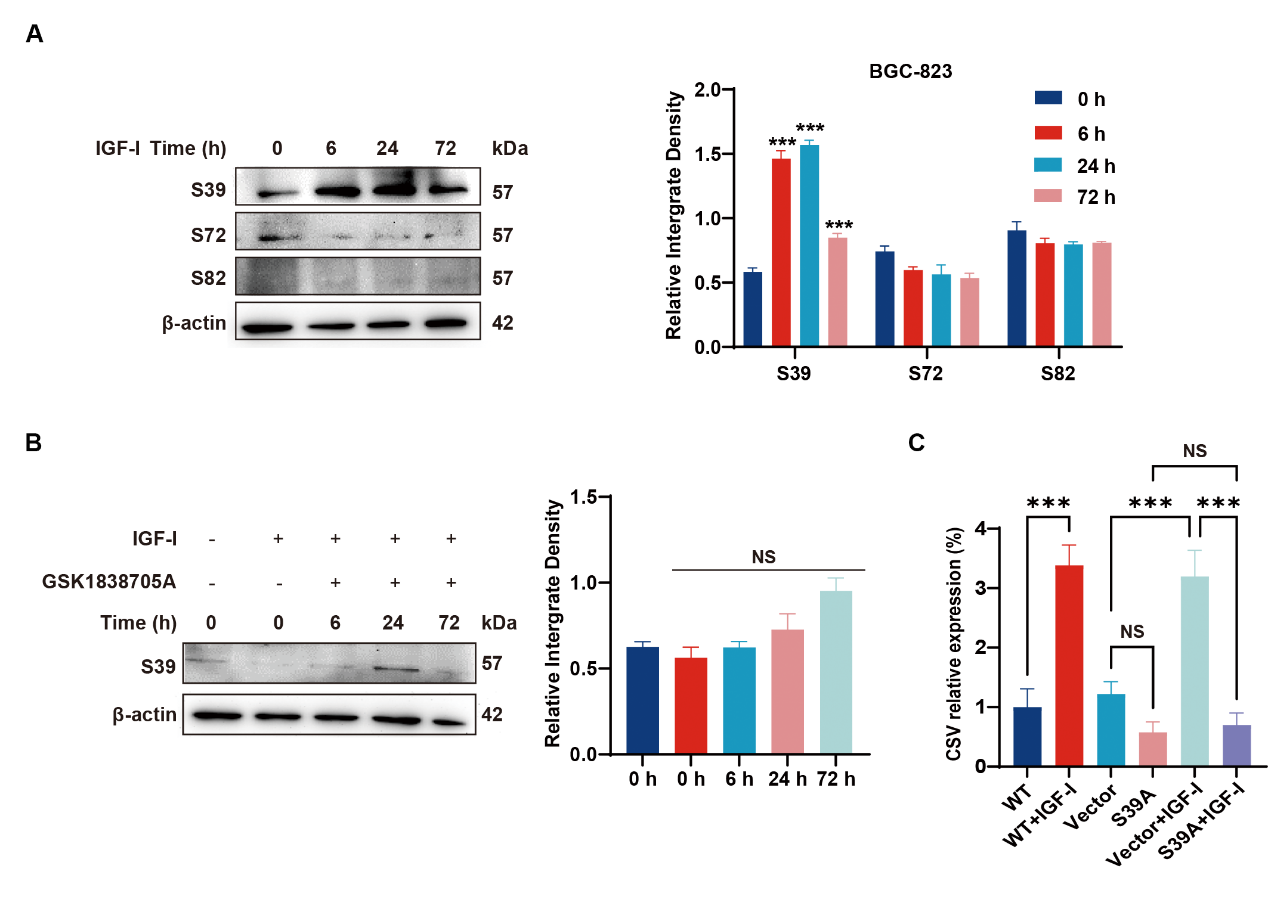


**Fig. S11: IGF-I induced the phosphorylation of vimentin in GC cells. (A)** GC cells were serum-starved, followed by treatment with 100 ng/mL IGF-I for 6, 24, or 72 hours. Phosphorylated vimentin levels (at serine residues S39, S72, S82) were measured by Western blot. **(B)** GC cells were serum-starved and subsequently treated with 100 ng/mL IGF-I, both alone and in combination with GSK 1838705A, for 6, 24, or 72 hours. Phosphorylated vimentin levels (S39) were assessed using Western blot assay. ***P < 0.001; NS = not significant. *(C) GC cells were transiently transfected with wild type or specific S39 mutant plasmid for 48 h followed by IGF-I stimulation for 48h, the expression levels of CSV were detected through flow cytometry. ***P < 0.001; NS = not significant.*

**
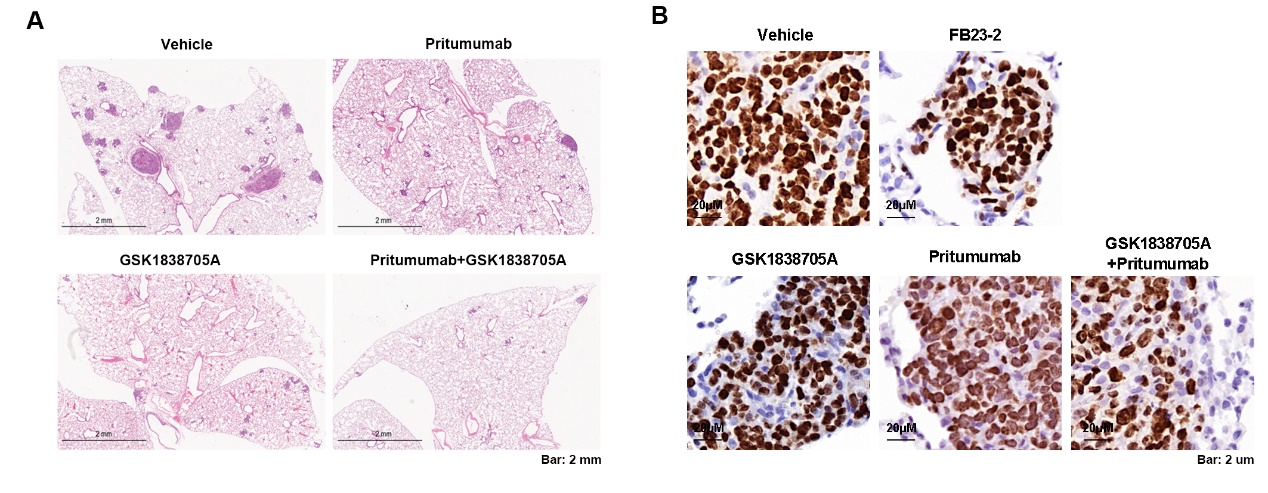
**

**Fig. S12: (A)** IHC staining of Ki67 expression of tumor lesions in different groups. Bar = 20 μm. **(B)** Representative H&E-stained sections of metastatic nodules in lung tissue from mouse model. Bar = 2 mm.
